# Head movement kinematics are altered during gaze stability exercises in vestibular schwannoma patients

**DOI:** 10.1038/s41598-021-86533-3

**Published:** 2021-03-30

**Authors:** Lin Wang, Omid A. Zobeiri, Jennifer L. Millar, Michael C. Schubert, Kathleen E. Cullen

**Affiliations:** 1 Department of Biomedical Engineering, Johns Hopkins University, Baltimore, USA; 2 Department of Biomedical Engineering, McGill University, Montreal, QC Canada; 3 Department of Physical Medicine and Rehabilitation, Johns Hopkins University School of Medicine, Baltimore, USA; 4 Department of Otolaryngology-Head and Neck Surgery, Johns Hopkins University School of Medicine, Baltimore, USA; 5 Department of Neuroscience, Johns Hopkins University School of Medicine, Baltimore, USA; 6 Kavli Neuroscience Discovery Institute, Johns Hopkins University, Baltimore, USA

**Keywords:** Regeneration and repair in the nervous system, Sensorimotor processing

## Abstract

Gaze stability is the ability of the eyes to fixate a stable point when the head is moving in space. Because gaze stability is impaired in peripheral vestibular loss patients, gaze stabilization exercises are often prescribed to facilitate compensation. However, both the assessment and prescription of these exercises are subjective. Accordingly, here we quantified head motion kinematics in patients with vestibular loss while they performed the standard of care gaze stability exercises, both before and after surgical deafferentation. We also correlate the head kinematic data with standard clinical outcome measures. Using inertial measurement units, we quantified head movements in patients as they transitioned through these two vestibular states characterized by different levels of peripheral damage. Comparison with age-matched healthy control subjects revealed that the same kinematic measurements were significantly abnormal in patients both pre- and post-surgery. Regardless of direction, patients took a longer time to move their heads during the exercises. Interestingly, these changes in kinematics suggest a strategy that existed preoperatively and remained symmetric after surgery although the patients then had complete unilateral vestibular loss. Further, we found that this kinematic assessment was a good predictor of clinical outcomes, and that pre-surgery clinical measures could predict post-surgery head kinematics. Thus, together, our results provide the first experimental evidence that patients show significant changes in head kinematics during gaze stability exercises, even prior to surgery. This suggests that early changes in head kinematic strategy due to significant but incomplete vestibular loss are already maladaptive as compared to controls.

## Introduction

Gaze stability is the ability of the eyes to fixate a stable point in the environment while the head is moving relative to space. The brain's capacity to provide stable gaze is essential in our daily lives. Notably, we experience rotational head velocities reaching ~ 450 °/s and linear head accelerations exceeding ~ 4 G during our everyday activities^1,2^. Additionally, during common behaviors such as walking and driving, we generate simultaneous rotational and translational head motion^1–4^. In this context, the vestibular system makes a vital contribution to gaze stability that enables us to see clearly during the dynamic and complex head movements elicited by such behaviors^5^. Accordingly, during head motion patients with vestibular hypofunction have poor gaze stability, for which the resulting blurred vision is associated with dizziness, fall risk, and low health related quality of life^6–8^.

A reduction in the efficacy of the vestibulo-ocular reflex (i.e., VOR) pathway underlies the poor gaze stability found in patients with vestibular hypofunction. Specifically, head motion information is encoded by afferents in the vestibular nerve, which in turn transmit the sensory feedback about our current head motion to the central neurons in the vestibular nuclei that mediate the vestibulo-ocular-reflex^9^. These central neurons project to extraocular motoneurons to generate the compensatory VOR eye movements that have been shown to provide gaze stability over the full frequency range of natural head movement behavior^10–12^, for which visually driven eye movements would be too slow. Because the marked impairment in the VOR of patients with unilateral or bilateral vestibular loss results in a reduction in gaze stability, current clinical practice guidelines (CPG) recommend gaze stabilization exercises as the critical component to rehabilitation efforts^13–16^. Typically, these exercises require patients to fixate on a visual target while moving their head horizontally or vertically, putatively to facilitate compensation that improves ability to stabilize their gaze during fast head movement. Improvements in gaze stability are thought to be largely mediated via recruitment of central compensatory mechanisms that improve VOR efficacy^17,18^. Additionally, the emergence of centrally programmed coupling of head motion and compensatory eye movements**,** as well as the upweighting from neck proprioceptive input^19–21^, likely contribute to compensation^18^. The upweighting of such extravestibular inputs occurs at the first stage of central vestibular processing in the vestibular nuclei to improve gaze stability following peripheral vestibular loss^22,23^.

Importantly however, to date, the recommendation and assessment of gaze stabilization exercises are not based on objective criteria. Instead, they follow the subjective assessment of the prescribing clinician^13^. In a recent study, Roller and Hall^24^ suggested the use of a metronome to ensure head velocities were in the ranges requiring a primary contribution from the VOR for gaze stability. However, no study has quantified head motion kinematics while performing gaze stability exercises. Thus, the goal of the current study was to quantify head motion kinematics in vestibular loss patients during standard vestibular rehabilitation gaze stability exercises, before and after surgical deafferentation, and to correlate kinematic data with standard clinical outcome measures. The quantifiable kinematic information captured via inertial measurement units (IMUs) in combination with commonly utilized clinical outcomes could enhance a clinician’s overall understanding of patient performance in a clinical setting, and potentially guide rehabilitation.

Accordingly, quantified head kinematics were measured using IMUs^25^ in vestibular schwannoma (VS) patients exposed to a single session of gaze stabilization exercises before and 6 weeks after surgical vestibular nerve deafferentation and compared with those of age-matched healthy control subjects. We found that the same kinematic measurements were abnormal in patients both pre- and post-surgery. Notably, across exercises, the most informative kinematic measures were (i) the average time required to finish one cycle of head motion (e.g., side to side, up and down), that is cycle duration, (ii) average of range of motion, and (iii) variability in the range of motion. Comparison of these kinematics measures with standard clinical measures revealed that cycle duration is a good predictor of our patients’ outcomes on various clinical measures [e.g., Dynamic Visual Acuity test (DVA), Timed up and go (TUG), and gait speed] both before and after surgery. Finally, we found that an overlapping subset of pre-surgery clinical measures (i.e., TUG, gait speed, and VOR gains) were well correlated with post-surgery kinematic measures of cycle duration, suggesting that patients’ vestibular impairment before the surgery was predictive of their head movement kinematics after the nerve resection. Our results provide the first experimental evidence that patients show significant changes in head kinematics during gaze stability exercises because of the presence of the tumor, prior to surgery. As a result, rehabilitation training prior to the surgery (i.e., “prehab”) may be advantageous.

## Methods

### Subjects

We recruited 18 patients with unilateral vestibular schwannoma that were scheduled for surgical resection. Of these, 9 patients completed all phases of the study (n = 9 males, mean 56.1 ± 15.7 years old, range 24–73 years old), where each patient was measured before and 6-weeks after the onset of surgery. We also recruited n = 9 age-matched healthy participants (8 males and 1 female mean 49.3 ± 15.0 years old, range 24–72 years old) with no history of otologic or neurologic disease. The study was approved by the Johns Hopkins University Institutional Review Board, and written informed consent was obtained from each individual. Pre-surgery measures were collected in an outpatient setting before (mean = 8 ± 13 days) the vestibular schwannoma tumor resection surgery. The post-surgery measures were collected at approximately 6 weeks (36–42 days) after the surgery. Both traditional clinical measures and kinematic measures of gaze stability exercises were collected at the same time points.

### Clinical measures

#### Dynamic visual acuity (DVA)

The DVA test measures the functional outcome of the patients' VOR during active head rotation. DVA was measured using a portable laptop and a motion sensor as developed^26^ and validated^27^ by Rine et al. The portable DVA was then normed in 3992 individuals^28^. We implemented a modified protocol per Millar et al.^29^. Specifically, a Samsung Galaxy Pro tablet (Seoul, South Korea) was used to present the visual stimuli and record the patients' static and dynamic acuity scores. Static visual acuity was measured first while the subject sat 200 cm from the tablet with their head still. Participants were required to distinguish one letter at a time presented on the tablet. The letter was randomly selected from ten optotypes (capital letters C D H K N O S R V Z). Visual acuity during active sinusoidal head rotations was then measured and scored separately for ipsi and contral-lesional head rotation. Each subject wore a single inertial measurement unit (IMU) (XSENS Technologies, Enschede, Netherlands) attached to a headband. This software generates the visual stimulus once the IMU has detected a head rotation with a velocity greater than 120 °/s. The scores were tabulated in the logarithm of the minimal angle resolution (LogMAR). Possible LogMAR scores ranged from − 0.3 to 1.7 (Snellen equivalent of 20/10 to 20/800). Corrected DVA scores were then calculated by subtracting the logMAR score of static visual acuity from the logMAR score of ipsilesional and contralesional DVA, respectively.

#### Timed up and go (TUG)

The TUG task measured each subject's ability to stand from sitting, walk 3 m and turn 180° before return to sitting position. Task performance was scored by measuring the time between when the subject's back left the chair to when their back touched the chair again. Each patient completed two TUG trials, turning ipsilesionally and contralesionally respectively when they passed the obstacle. Scores on the TUG > 11.1 s correlate with reports of falls in persons with vestibular dysfunction^30^.

#### Gait speed

The Ten Meter Walk Test (10MWT) measured the subject's self-selected comfortable walking speed over a 10 m distance. The patients started and stopped at least 2 m beyond the 10 m range to ensure the measured gait speed did not include the acceleration or deceleration phases of the locomotion. Their average gait speed was computed over the 10 m distance.

#### Functional gait assessment (FGA)

The FGA comprises 10 unique walking exercises: (1) Gait on a level surface, (2) Change in gait speed, (3) Gait with horizontal head turns, (4) Gait with vertical head turns, (5) Gait and Pivot turn, (6) Step over obstacle, (7) Gait with narrow base of support, (8) Gait with eyes closed, (9) Ambulating backwards, and (10) Steps. An experienced clinician scored each task between 0 and 3 points with 0 indicating severe impairment and 3 indicating normal ambulation. FGA scores less than 22 (30 total) are predictive of falls in older adults^31^.

### Physiological measures

#### Video head impulse test (vHIT)

The vHIT (ICS Otometrics, Natus Medical Incorporated, Denmark) measures VOR gain (eye velocity/head velocity) during passive head movement. Subjects were seated 1 m from a stationary visual target, in room light. At least 12 passive head rotations were performed in both directions of three planes parallel to the three pairs of semicircular canals: horizontal, right anterior/left posterior (RALP) and left anterior/right posterior (LARP). Right eye and head velocity were sampled at 220 Hz. vHIT traces were deleted if the eye velocity trace preceded head velocity, if the head velocity was below 100 °/s, or if the passive head rotation trace did not match the acceleration profile suggested by the manufacturer. VOR gain values within 0.8–1.2 with standard deviation < 0.12 were considered normal^29,32,33^.

### Subjective measures

#### Dizziness handicap inventory (DHI)

The DHI is a subjective measure that scores the impact of dizziness or unsteadiness on quality of life. The scale consists of 25 items in functional, emotional, and physical domains, with a total score of 0–100.

#### Activities-specific balance confidence scale (ABC)

The ABC scale is a self-report measure of balance confidence^34^. The subjective measure consists of 16 self-report items in which subjects rate their confidence of not losing balance while performing various daily activities from 0 (no confidence) to 100 (complete confidence). Previous studies suggested that the ABC score is an accurate indicator of fall risk among patients with vestibular disorders^35^.

#### Headache impact test

The headache impact test measures the impact headaches have on a subject's ability to function in daily life^36^. The scale consists of 6 items in which patients report how often (never-rarely-sometimes-very often-always) headache affects their daily activities.

#### Beck anxiety inventory (BAI)

The BAI is a self-report measure of anxiety with 21 items in which subjects rate their anxiety from: Not at all (0), Mildly (1), Moderately (2), and Severely (4)^37^. The total score is the sum of the 21 items with a score of 0–21 indicating low anxiety, 22–35 indicating moderate anxiety, and scores above 36 indicating a potentially concerning level of anxiety. In the current study, the patients were instructed to rate their anxiety that is only related to the symptoms caused by the vestibular schwannoma and its resection.

### Kinematic measurements of gaze stability exercises

Patients were instructed to complete 6 gaze stabilization exercises during which they generated active head rotations while fixating their gaze on an earth-stationary visual target. The target was an inch-sized letter "X" printed on a small piece of paper. These 6 exercises were varied based on two factors: (1) the direction of head motion: yaw (horizontal) or pitch (vertical), and (2) the placement of the target: fixated on a wall 1 m away (Table 1: exercise 1 and 2), hand-held (Table 1: exercise 3 and 4) and fixated on a wall in 2 m distance (Table 1: exercise 5 and 6). Subjects were instructed to continuously move their head (i.e., side to side or up and down) for 30 s at the highest velocity possible provided the target did not blur. During each exercise, the subject’s angular head velocity was recorded using a small (51 mm × 34 mm × 14 mm) MEMS sensor (Shimmer3 IMU, Shimmer Research, Dublin, Ireland) that was securely and comfortably attached to the back of patients' head using an elastic head band. The data were sampled at 500 Hz and recorded on a built-in micro SD card. Kinematic measurements were calculated based on the head angular velocity data, focusing on the dimension aligned with the direction of head motion during the gaze stabilization exercise (e.g., yaw in horizontal gaze stabilization exercises and pitch in vertical gaze stabilization exercises were analyzed respectively). Specifically, we identified each repetition as the instructed head movement. Each of these 'cycles' was defined as the head moving from one end (i.e., right or up) to the other end (i.e., left or down).

**Table 1:** A list of the 6 gaze stabilization exercises used in the current study.

Standing gaze stabilization exercises			
Exercise number	Head movement direction	Target location	Distance (m)
1	YAW	On wall	1
2	PITCH	On wall	1
3	YAW	In hand	1
4	PITCH	In hand	1
5	YAW	On wall	2
6	PITCH	On wall	2

The exercises vary in direction, target fixation and the distance between the patient and the target. Target on wall means the target was attached to a wall whereas target in hand means the target was held by the patient with their dominant hand at 90° shoulder flexion with full arm extension.

For yaw exercises (i.e., exercises 1, 3, and 5), we assigned the head movement cycles toward each side uniquely as ipsi- and contralesional based on the side of the tumor. For healthy controls, the right side was considered as ipsilesional. For each cycle we computed three kinematic measures: (i) Peak velocity: the highest rotational head velocity reached within the cycle. (ii) Cycle duration: the time spent to finish each cycle. (iii) Movement range: the range of head rotation in each cycle computed by integrating the rotational head velocity. Then we computed the mean and coefficient of variation (CV) of these 3 kinematic measures across all cycles for both directions of movement during each exercise (i.e., left and right/up and down). Finally, to obtain the asymmetry measures, we first computed the mean and coefficient of variation for each side of movement separately, then divided these values for ipsilesional or up sides by the contralesional or down sides for yaw and pitch head movement exercises, respectively.

In addition, kinematic score was computed based on three kinematic measures: (1) mean cycle duration, (2) mean movement range, and (3) coefficient of variation of movement range. First, each of these measures was normalized by a linear transformation of mean ± 2SD to a number between 0 and 100 (i.e., normalized mean = 50 and normalized SD = 25). Numbers outside the 0–100 range were then projected to the closest number within this range (i.e., either 0 or 100). The average of three normalized numbers across all selected gaze stabilization exercises was used as the kinematic score.

### Statistics

We performed non-parametric paired sample permutation (re-randomization) tests for all comparisons between kinematic and clinical measures from the vestibular patients (pre- and postoperative) and age-matched healthy controls. Specifically, p-values were computed obtaining the test statistics for 2000 randomized rearrangements of the observed data points. We also computed the Pearson correlation coefficients and p-values of kinematic and clinical measures using Pearson correlation. To examine whether trends were consistent across a number of exercises we examined (i) whether correlations are significant (p < 0.05) for the majority of exercises and (ii) whether, for all significant correlations the relationship had the same sign (i.e., correlation were consistently positive/negative across exercises). Throughout the text, values are expressed as mean ± 1 SD and significance is reported at p < 0.05. All data processing and statistical tests were performed using MATLAB (The MathWorks, Inc., Natick, Massachusetts, United States).

## Results

### Clinical measurements are abnormal in patients both pre- and post-surgery. Post-surgery measures of gaze stability (DVA and VOR) worsen

#### Pre-operative patients versus healthy controls

**Figure 1:**
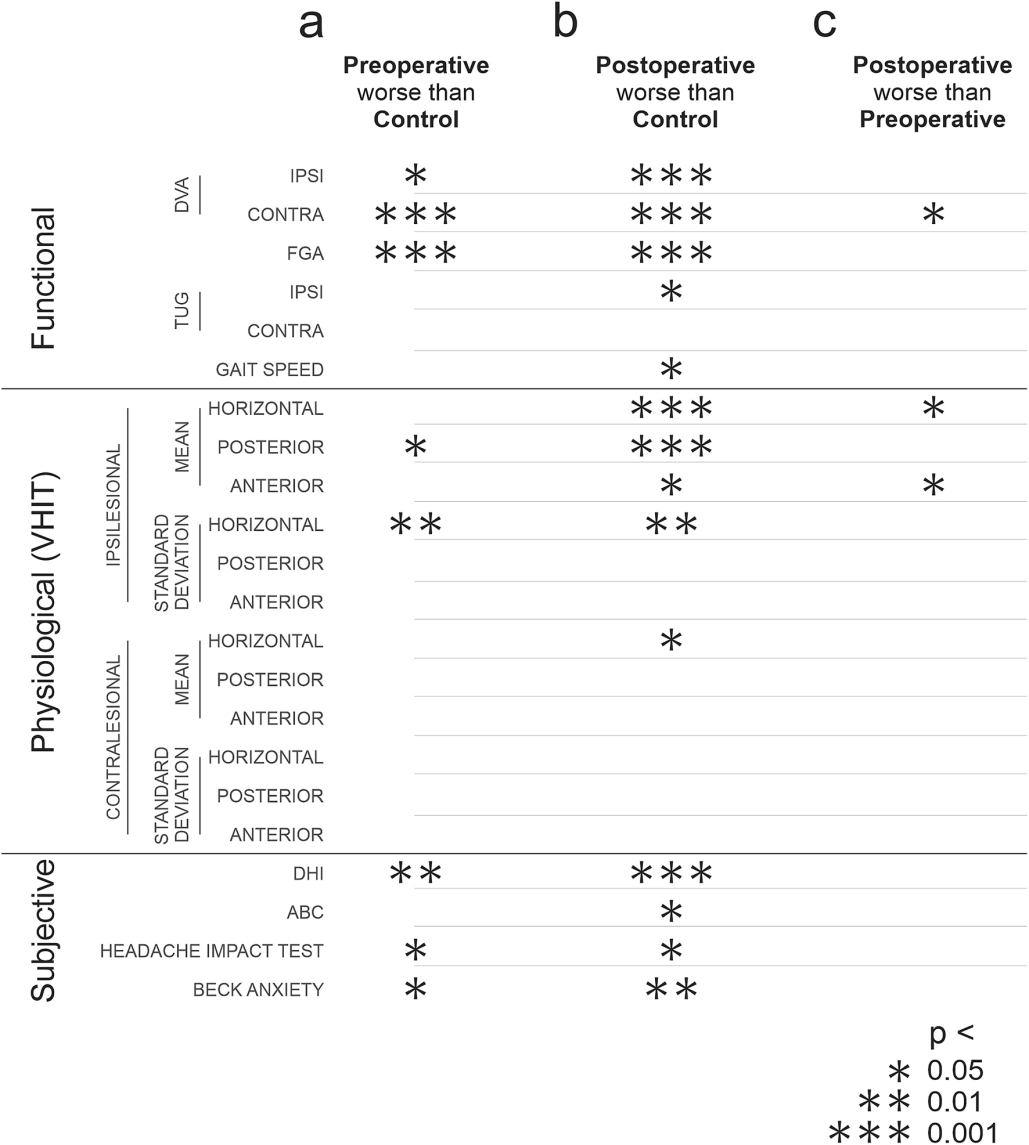
Comparison of clinical measurements across unique domains (clinical, physiologic, subjective) between (**a**) pre-surgery patients and healthy controls, (**b**) post-surgery patients and healthy controls, (**c**) post-surgery patients and pre-surgery patients. Asterisks indicate differences at three significance levels (*0.05, **0.01. ***0.001). DVA, dynamic visual acuity; FGA, functional gait assessment; TUG, timed up and go; vHIT, video head impulse test; DHI, dizziness handicap inventory; ABC, activities-specific balance confidence scale.

Figure 1a shows the comparison between clinical, physiological, and subjective measures obtained for patients before surgery (i.e., pre-surgery testing) and healthy controls. First, the comparison of preoperative clinical measures revealed that patients had significantly worse DVA scores for both ipsilesional and contralesional head rotations. Additionally, the FGA scores for the patient subjects were abnormal pre-operatively. In contrast, TUG and gait speed were comparable between the groups (Fig. 1a)**.** Next, the comparison of physiological measures between these two subject groups revealed that the mean VOR gain for ipsilesional head rotations of the posterior semicircular canal, as well as the variability (SD) of ipsilesional horizontal head rotations were much worse pre-operatively than healthy controls. Finally, within the domains of subjective experience, the DHI, headache impact test, and BAI scores also show pre-surgery impairment in the patient subjects. Pre-surgery ABC scores were however comparable to the scores from healthy controls.

#### Post-operative patients versus health controls

Figure 1b illustrates the difference between clinical, physiological, and subjective measures obtained for post-surgery patients and healthy controls. The comparison of clinical measures revealed that post-surgery patients had worse ipsilesional and contralesional DVA, FGA scores, ipsilesional TUG and gait speed scores than controls. Further, our comparison of physiological measures revealed that the average post-operative patients’ VOR gain (as measured per vHIT) was worse than healthy controls in each of the three semicircular canals for ipsilesional head rotations, and also for contralesional rotation in the horizontal semicircular canal (Fig. 1b). The standard deviation of the post-operative patients’ VOR gain in the ipsilesional horizontal semicircular canal was also worse than controls. Finally, the comparison of subjective measures revealed that the post-operative patients displayed worse DHI, ABC, headache impact test and BAI scores than healthy controls (Fig. 1b).

#### Pre-operative versus post-operative patients

Figure 1c illustrates the direct comparison between the preoperative and postoperative data obtained for our patient group. Note that only contralesional DVA and average VOR gain in the ipsilesional horizontal and anterior semicircular canals were significantly worse 6 weeks after surgery compared to prior to surgery.

### Kinematic measurements are abnormal in patients both pre- and post-surgery compared to healthy controls. No significant improvement at 6 weeks post-surgery

**Figure 2:**
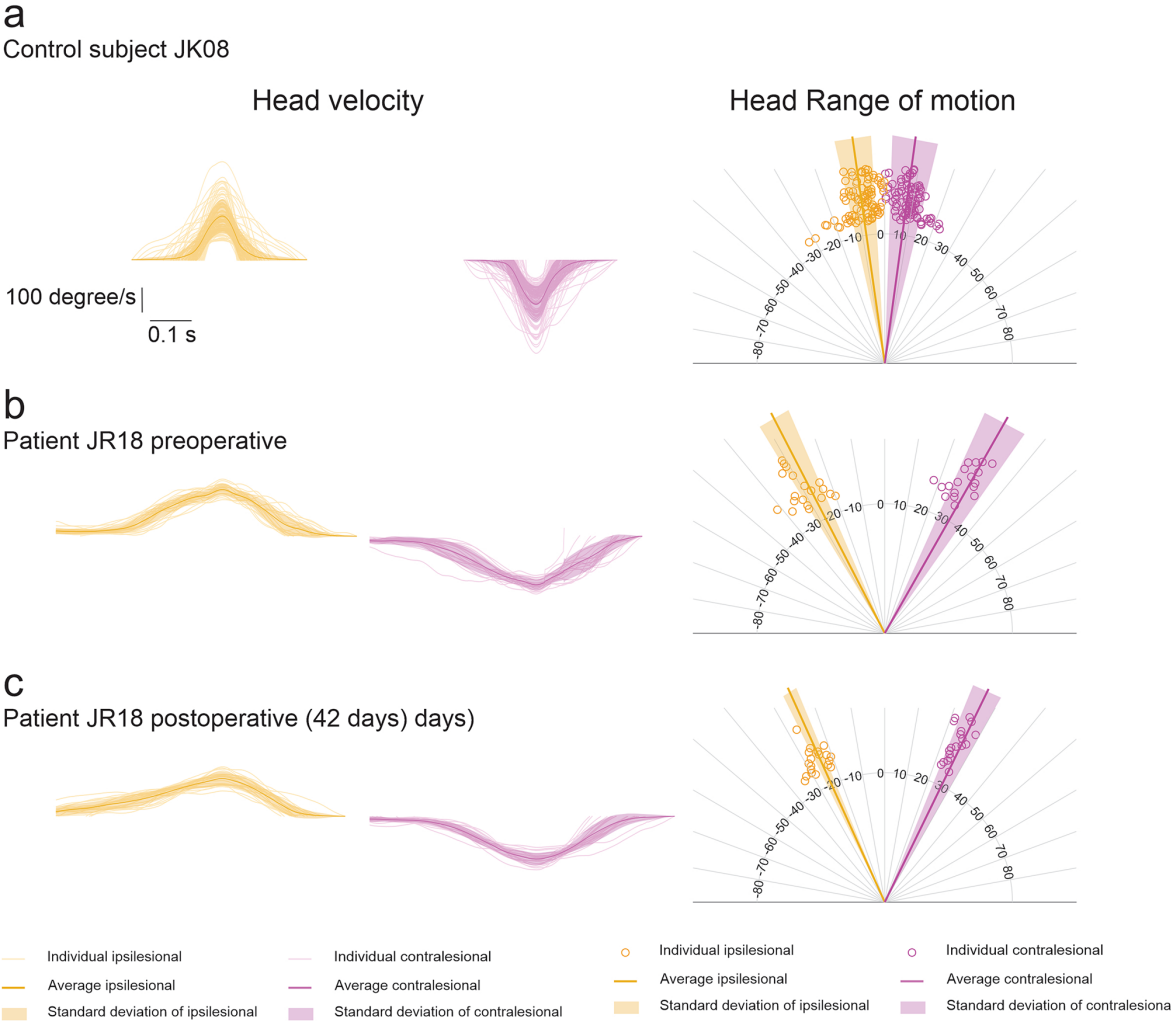
Example data from (**a**) one healthy control and (**b**) pre-surgery and (**c**) post-surgery testing from one patient in the standing X1 gaze stabilization exercise with horizontal head rotation. The target was 1 m away from the subject and fixed on the wall. The left panels show the head velocity traces from each head rotation cycle superimposed with the mean and standard deviation of head velocity. The right panels show the range of motion of each head rotation as well as the mean and standard deviation of the range of motion. Orange traces and circles indicate head rotation ipsilateral to the lesion side and magenta traces and circles indicate head rotation contralateral to the lesion side.

We next quantified head kinematic data recorded during gaze stabilization exercises in the same subjects across time points. Figure 2 shows example data from a typical healthy control (Fig. 2a) and a typical patient pre- and post-surgery (Fig. 2b,c respectively) during the standing X1 gaze stabilization exercise involving horizontal head rotations with a visual target fixed on the wall 1 m away from the subject (see, Table 1, exercise 1). One clear feature of the example data is while patients and healthy control subjects generated head movement that reached comparable peak velocities, cycle durations were significantly longer (e.g., + 0.55 s; 230% during exercise 1), and the range of motion was greater and less variable for patients at both time points.

**Figure 3:**
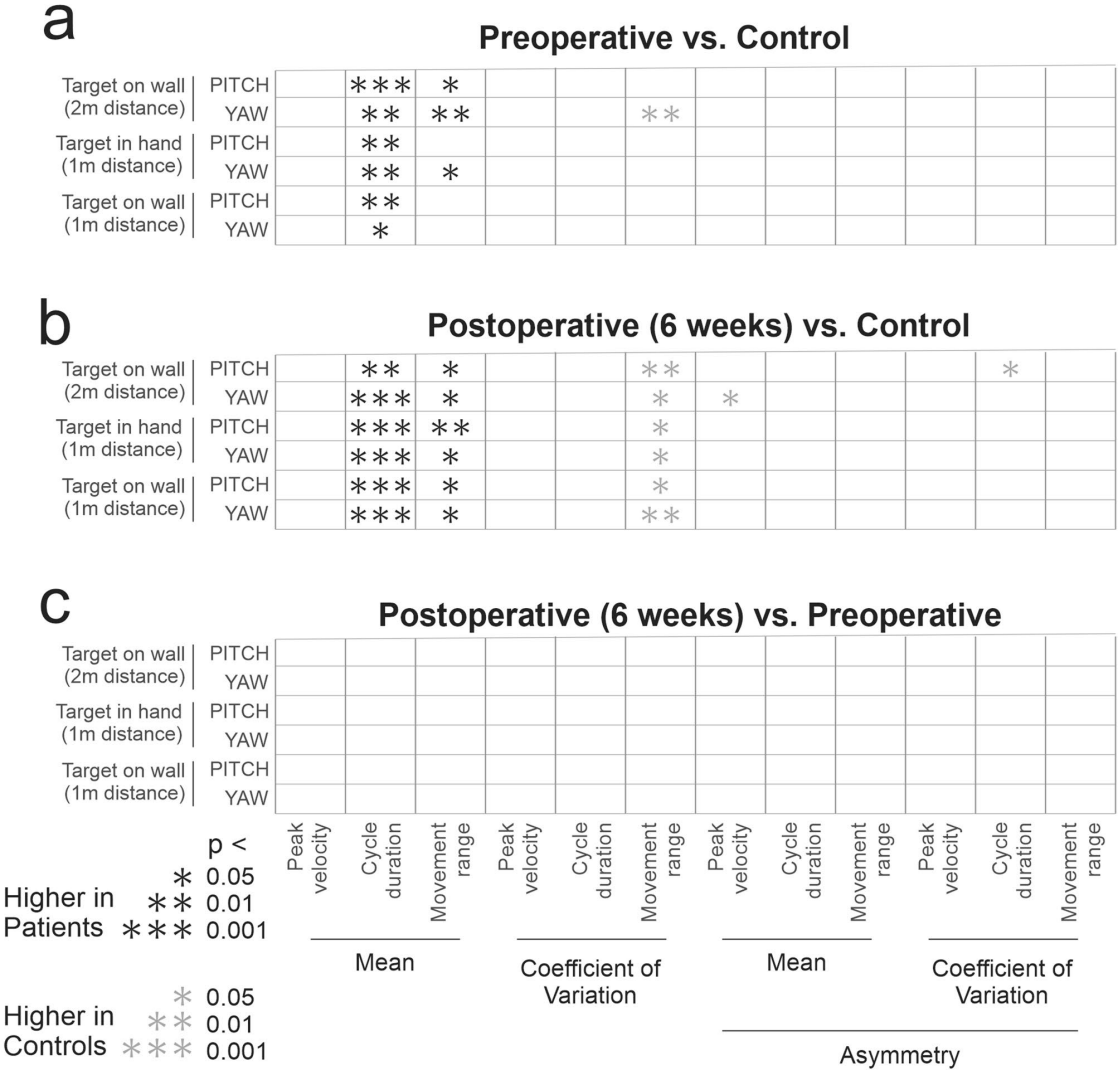
Comparison of kinematic measurements between (**a**) pre-surgery patients and healthy controls, (**b**) post-surgery patients and healthy controls, (**c**) pre-surgery patients and post-surgery patients. The kinematic measurements are arranged horizontally. The 6 exercises are arranged vertically. Asterisks indicate differences at three significance level (*0.05, **0.01. ***0.001). Black asterisks indicate the first group as indicated in the title had a larger value than the second group and grey asterisks indicate the first group had a smaller value than the second group.

The observations shown above for our example subjects in Fig. 2 are summarized for our populations of control and patient subjects in Fig. 3, and Table 2. Specifically, Fig. 3a compares the head kinematic measures between pre-surgery patients and healthy controls. As illustrated in this figure, three types of measurements were the most effective in demonstrating impairments in patient performance: (i) mean cycle duration, (ii) mean movement range, and (iii) coefficient of variation of movement range. Specifically, patients took longer to finish one head movement cycle than controls for all 6 exercises. They also had a larger range of head rotations than controls for 3 out of the 6 exercises. Finally, the range of head movement made by patients was less variable than controls for 1 out of 6 exercises. In contrast, we did not find any significant differences for our head velocity and asymmetry measures. Figure 3b compares the head kinematic measures between post-surgery patients and healthy controls using an analogous structure. Similar to their pre-surgery measurements, patients also showed a longer cycle duration, as well as a larger and less variable range of head movement for all 6 exercises. Interestingly, as shown in Fig. 3c, the comparison for our patient group before and after surgery showed little difference between pre- and post-surgery measurements. Overall, our findings show that kinematic measures in patients were altered relative to controls even before the surgery, due to the impact of their tumor. Further, our results show that these differences were maintained over at least a 6-week duration from the surgical deafferentation. Table 2 reports the means and standard deviations for the kinematic measures described above, for which there were generally significant differences between groups (Fig. 3), specifically, the mean cycle durations, mean movement ranges, and coefficient of variation of movement range.

**Table 2:** Means ± SD of the six kinematic measures that demonstrated the most significant difference between vestibular patients (pre- and postoperative) and healthy controls.

	Healthy controls			Preoperative			Postoperative		
	Mean cycle duration (s)	Mean movement range (°)	CV movement range	Mean cycle duration (s)	Mean movement range (°)	CV movement range	Mean cycle duration (s)	Mean movement range (°)	CV movement range
Exercise 1 YAW Target on wall 1 m distance	0.24 ± 0.07	17 ± 8.1	0.32 ± 0.16	0.68 ± 0.59	26 ± 10	0.19 ± 0.11	0.79 ± 0.53	26 ± 9.1	0.14 ± 0.06
Exercise 2 PITCH Target on wall 1 m distance	0.22 ± 0.05	8.7 ± 4.8	0.34 ± 0.2	0.62 ± 0.57	15 ± 9.2	0.26 ± 0.16	0.68 ± 0.48	17 ± 6.5	0.17 ± 0.09
Exercise 3 YAW Target in hand 1 m distance	0.23 ± 0.05	15 ± 7.4	0.32 ± 0.15	0.64 ± 0.53	24 ± 10	0.26 ± 0.19	0.65 ± 0.44	24 ± 8.1	0.17 ± 0.11
Exercise 4 PITCH Target in hand 1 m distance	0.23 ± 0.07	8 ± 5.4	0.39 ± 0.18	0.6 ± 0.49	15 ± 8.5	0.28 ± 0.15	0.68 ± 0.48	18 ± 7.3	0.21 ± 0.15
Exercise 5 YAW Target on wall 2 m distance	0.23 ± 0.04	12 ± 4.7	0.33 ± 0.13	0.68 ± 0.52	25 ± 10	0.16 ± 0.09	0.78 ± 0.69	23 ± 10	0.17 ± 0.11
Exercise 6 PITCH Target on wall 2 m distance	0.23 ± 0.06	7.6 ± 4.9	0.4 ± 0.2	0.64 ± 0.52	15 ± 8.2	0.24 ± 0.11	0.74 ± 0.62	16 ± 7.5	0.17 ± 0.12

Each row corresponds a gaze stabilization task.

### Pre-surgery head movement cycle duration correlates with multiple clinical and physiological pre-surgery measurements

**Figure 4:**
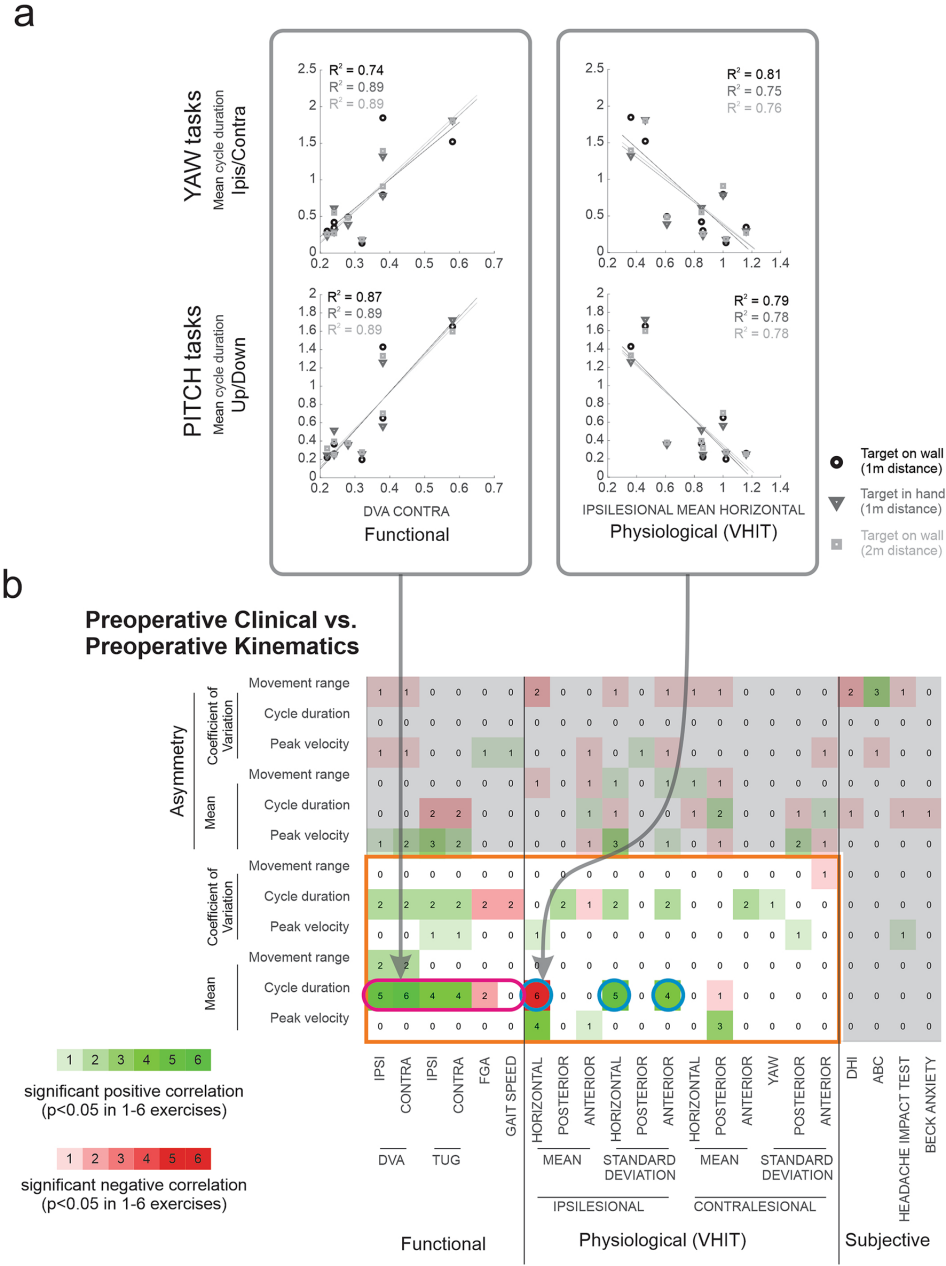
(**a**) Scatter plots showing the correlation between cycle duration and contralesional DVA score (left panel) and between cycle duration and ipsilesional horizontal semicircular canal VOR gain (right panel) in all the 6 exercises. (**b**) Correlations between pre-surgery kinematic measurements and pre-surgery clinical measurements. Green squares indicate positive correlations and red squares indicate negative correlations. Brightness and number in the square indicate the number of exercises (1–6) showing a significant correlation (p < 0.05).

We asked whether there was any relationship between pre-surgery kinematic and clinical measures. Figure 4a illustrates the significant correlations between the mean cycle duration and two clinical measures during all six exercises. Specifically, preoperative patients who took longer to finish head movement cycles, had higher contralesional DVA scores (Fig. 4a; left column) and lower ipsilesional mean VOR gain in the horizontal plane (Fig. 4a; right column). Figure 4b summarized the relationships between all pre-surgery kinematic (vertical axis) vs. clinical (horizontal axis) measures. Each number indicates the number of exercises in which we found significant correlations. The green and red background colors correspond to positive and negative correlations, respectively. Most notably, cycle duration was strongly correlated with multiple clinical measurements in most of the 6 exercises (i.e., 4–6), suggesting that cycle duration is a good indicator of patient's pre-surgical performance. Specifically, cycle duration is correlated with DVA scores on both sides, and completion time of TUG for both ipsi and contralesional trials (Fig. 4b, magenta oval). Further, in a majority of exercises, the cycle duration correlated with mean VOR gain in the ipsilesional horizontal semicircular canal plane and standard deviations of VOR gain in the ipsilesional horizontal and anterior semicircular canal planes (Fig. 4b, blue circles). Note, a complete set of tables showing the corresponding correlations between kinematic and clinical measures for each individual exercise is provided in the Supplementary Information (Supplementary Tables 1–6). These results suggest that patients who need a longer time to finish a head rotation cycle also have impaired dynamic visual acuity, VOR, and gait velocity. It further confirms that gaze stability is a basic function that supports daily activities such as walking.

In contrast, none of our subjective self-report measures (i.e., DHI, ABC, headache impact test, and BAI) demonstrated significant correlations with cycle duration (i.e., time to move head side to side, up and down) in any of the exercises. This suggests that subjective measurements are less sensitive in capturing patients' impairment than objective measurements. Finally, in addition to cycle duration, we found that mean peak velocity was positively correlated with mean VOR gain in the ipsilesional horizontal semicircular canal plane and with the standard deviation of VOR gain in the contralesional posterior semicircular canal plane in 4 and 3 of the 6 exercises, respectively. This result is not surprising given that patients with a more compromised VOR would experience more significant retinal blur during head movements. Thus, to minimize their gaze stability, such patients would be likely to generate lower velocity head movements. Other kinematic measurements did not show consistent correlations with clinical measurements across exercises.

### Post-surgery head movement cycle duration was correlated with multiple clinical but not physiological post-surgery measurements

**Figure 5:**
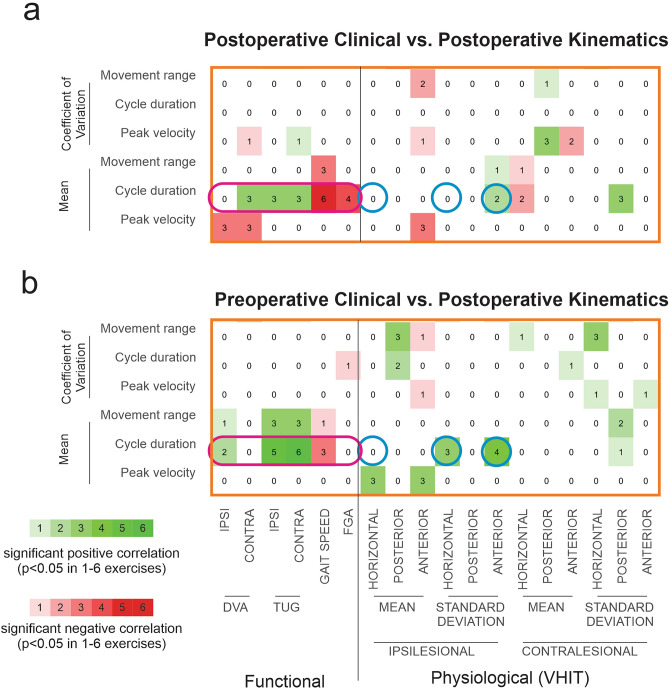
Correlations between post-surgery kinematic measurements and (**a**) post- and (**b**) pre-surgery clinical measurements. Green squares indicate positive correlations and red squares indicate negative correlations. Brightness and number in the square indicate the number of exercises (1–6) showing a significant correlation (p < 0.05).

We next asked whether the relationships between kinematic and clinical measures observed in our patients before surgery (Fig. 4) were also observed post-surgery. Figure 5a illustrates the significance of the correlations between post-surgery measurements across exercises (corresponds to the large area in Fig. 4 outlined in orange). Interestingly, as observed in the pre-surgery state, head movement cycle duration was again the most informative kinematic measure in that it correlated with several clinical measures in most exercises (Fig. 5a, magenta oval). Specifically, as observed in the pre-surgery state, head movement cycle duration was correlated with clinical measures including contralesional DVA, both ipsilesional and contralesional TUG times, and gait speed. In addition, cycle duration was correlated with the FGA score in the post-surgery but not pre-surgery state (compare the right cell in magenta ovals, Fig. 4b vs. Fig. 5a). In contrast, no consistent correlations were observed between kinematic measures and physiological measures after the surgery. In particular, the average and variability of ipsilesional horizontal semicircular canal VOR gain, showed a correlation with cycle duration before but not after surgery (Fig. 4b vs. Fig. 5a, blue circles). Finally, we note that our subjective self-report measurements (i.e., DHI, ABC, headache impact test and BAI) again did not demonstrate significant correlations with cycle duration in any of the exercises (not shown).

Taken together, these results indicate that the patients who required the most time to complete a head rotation cycle also demonstrated impaired gait characterized by slower walking. Further, due to nerve resection, the ipsilesional DVA score and VOR gains were severely impaired across all patients (i.e., Fig. 1), and as a result correlations between these physiological measures and kinematic measures were eliminated.

### Pre-surgery clinical measures can predict post-surgery kinematic measures

Finally, we asked whether we could leverage our quantification of clinical and physiological measures, in the pre-surgical state to predict the kinematics of patient head movements, during the gaze stabilization exercises after the surgery. This analysis is shown in Fig. 5a, which again reports the number of significant correlations across exercises. Comparison between pre-surgery clinical and physiological measurements and post-surgery kinematic measures revealed a consistent relationship between pre-surgery clinical measures (i.e., TUG and gait speed) and post-surgery mean cycle duration (Fig. 5b, magenta oval) and mean movement range kinematic measures. Further, pre-surgery variability of the ipsilesional VOR gain in horizontal and anterior semicircular canal planes correlated with mean cycle duration during most exercises (Fig. 5b, center and right blue circles). However, in contrast to the corresponding pre-surgery correlation results, the average pre-surgery ipsilesional VOR gain in the horizontal plane was not correlated with the mean cycle duration post-surgery (compare left blue circle in Fig. 4b and 5b). Again, a complete set of tables showing the corresponding correlations between kinematic and clinical measures for each individual exercise is provided in the Supplementary (Supplementary Tables 7–18). Overall, these results suggest the movement behavior of patients with vestibular schwannoma 6 weeks after complete unilateral vestibular loss can be partially predicted from their functional state before the surgical resection of the vestibular nerve.

### Quantifying the global change in kinematics in VS patients before and after vestibular neurectomy based on the most informative kinematic parameters

**Figure 6:**
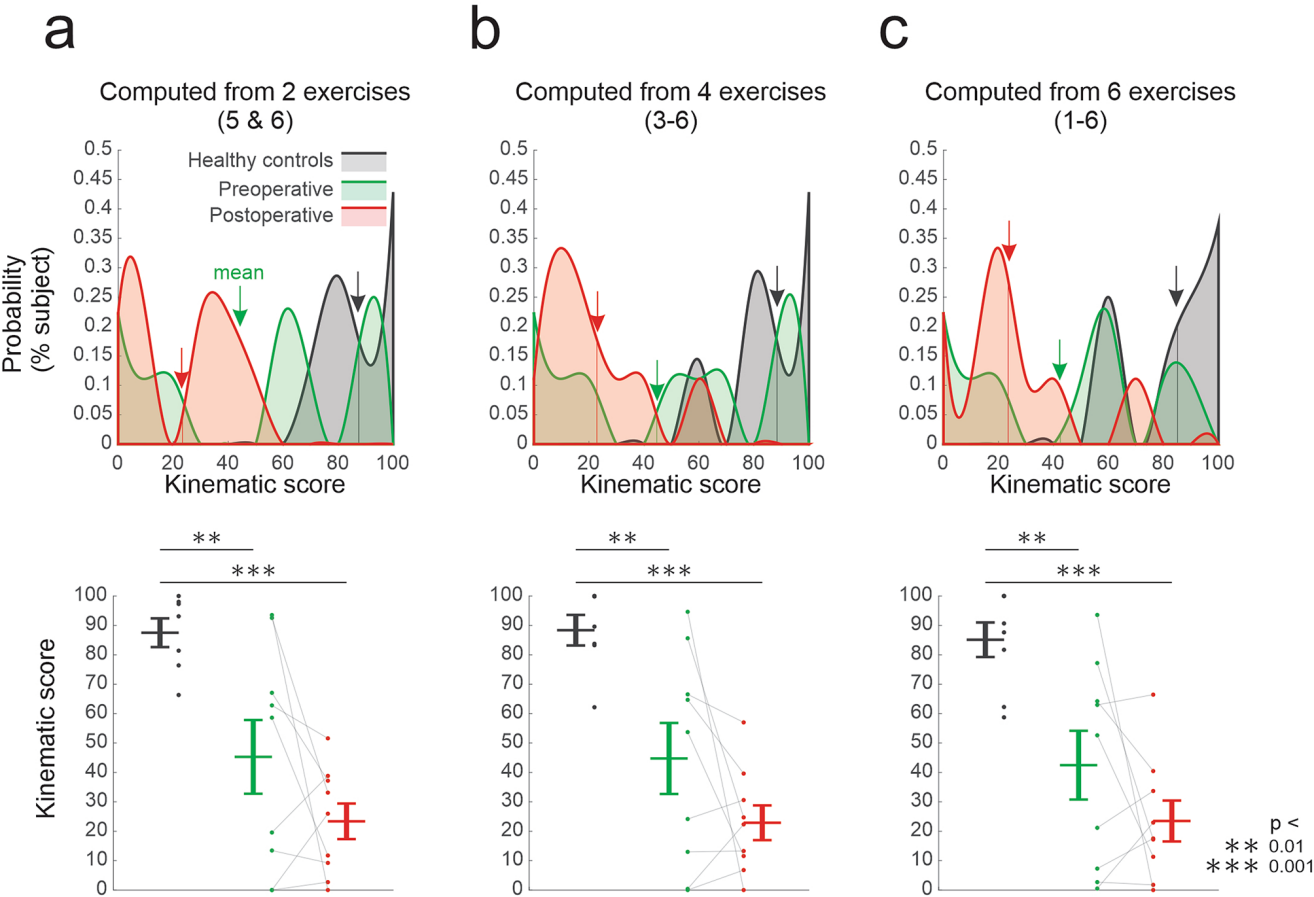
Comparison kinematic scores computed for (**a**) the 2 most informative gaze stabilization exercises (standing far), (**b**) 4 gaze stabilization exercises (3 to 6), and (**c**) across all 6 exercises. (**a**–**c**) Top: Probability distributions of the kinematic scores computed for healthy controls (black), pre-operatively (green), and post-op patients (red). Arrows indicate the average values. Bottom: Comparison of the kinematic scores of healthy controls versus unilateral vestibular patients. Vertical lines correspond to the mean ± SEM of the kinematic score for each group, while the kinematic scores for individual subjects are illustrated as points. Asterisks denote significant difference between healthy controls and patients (*p < 0.05, **p < 0.01, ***p < 0.001).

Our results demonstrate that measuring specific head movement kinematics during gaze stabilization exercises—in particular mean head movement cycle duration, as well as the mean and coefficient of variation of movement range—can provide valuable information. These results raise the question of whether it is feasible to collapse the wide range of measures we made into a global kinematic score that would be useful to clinicians who are evaluating VS patients before and after tumor resection. To this end, we computed a single score (see “Methods”) based on the three kinematic measures that were most informative (i.e., consistently displaying significant differences relative to healthy controls) in our results: mean cycle duration, mean movement range, and coefficient of variation of movement range (Fig. 3, Table 2). We compared this score when it was computed for (i) the 2 most informative gaze stabilization exercises (i.e., those for which we found the most significant differences between patients and healthy controls) (Fig. 6a; exercises 5 and 6), (ii) these exercises were then combined with the next 2 most informative gaze stabilization exercises (Fig. 6b; exercises 3 to 6), and (iii) finally across all 6 exercises (Fig. 6c), with the score scaled (see “Methods”) over a range from 0 (most abnormal) to 100 (normal).

Figure 6 illustrates that all three of these computations yielded similar results, with healthy controls scoring closest to 100%, followed by preoperative patients, and then acute postoperative patients. Further, controls were significantly different than both preoperative patients (p < 0.05) and postoperative patients p < 0.001) regardless of the computation. Notably, we observed inter-subject variability in the kinematic-based score for preoperative patients (Fig. 6; green shades) comprising high scores (> 50) that are comparable with healthy controls but also lower scores (< 50) comparable with those from the postoperative patients. Thus, our analysis shows the robustness of a computation based on specific kinematic measures. Also, our results highlight the potential utility of focusing on a subset of gaze stabilization exercises (i.e., exercise 5&6) in the assessment of head kinematics post-operatively. To develop the optimal approach to generate a single “kinematic score” from head kinematic data obtained during the gaze stabilization exercises, however, further algorithm development and testing utilizing a larger dataset is required and is an interesting direction for future work in this field.

## Discussion

In this study, we investigated the hypothesis that head motion kinematics during gaze stabilization exercises are significantly altered as a result of peripheral vestibular loss. To do this we quantified head motion kinematics in patients with VS before and 6 weeks after unilateral vestibular resection. We found several interesting results that have potential clinical implications. First, patients showed altered kinematics relative to age-matched control subjects before the surgery. Interestingly, (i) the same kinematic measurements that were abnormal in patients before surgery (i.e., average of head movement cycle duration, average of range of motion, and variability in the range of motion) remained abnormal post-surgery, and (ii) changes in these kinematic measures were symmetric. Second, the average of cycle duration in particular was a good predictor of patient’s clinical measures (e.g., DVA, TUG time, and gait speed), both before and after the surgery. Third, an overlapping subset of pre-surgery clinical measures (i.e., TUG, gait speed, and VOR gains) were predictive of their head movement kinematics after the nerve resection. Thus, taken together, our findings suggest that such early changes in head motion strategy are maladaptive as compared with healthy controls, and providing rehabilitation prior to the surgery (i.e., prehab) could be beneficial.

### Standard of care and implications for ‘prehab’

The current standard of care for patients with UVD is to provide rehabilitation only after the surgery is complete. However, studies have shown that pre-surgical intervention can reduce patients' symptoms and improve their short-term and long-term recovery^38,39^. Our data reveal that pre-operatively, patients behave differently from healthy controls, suggesting that rehabilitation efforts may be valuable to offer before surgery. In particular, our pre-surgery measures revealed that patients had a longer head movement cycle duration, a larger range of motion, and a smaller variability of motion range compared to healthy controls. Differences were also observed in several of the traditional clinical measurements including DVA, vHIT, DHI, headache impact test, BAI and FGA scores. Furthermore, we found little difference between pre-surgery and post-surgery kinematic measures. Most of the differences observed relative to healthy controls persisted and at 6 weeks post-surgery patients were still abnormal. We did not control for rehabilitation efforts in the present study, and therefore whether these results would differ if rehabilitation were explicitly controlled remains to be examined. Nonetheless our results are surprising given schwannomas tend to grow slowly^40^, theoretically allowing time for central compensation—in which case one might expect patients to perform normally pre-operatively^41,42^. Our data suggest pre-surgical rehabilitation may be beneficial, which is not currently considered as standard of care.

Our results further show that ipsilesional kinematic measurements were comparable to contralesional kinematic measurements in both pre-surgical and post-surgical patients. None of the asymmetry measurements were significantly different from controls. Results from DVA test also show that both ipsi and contralesional DVA in pre- and post-surgery patients are worse than controls. In contrast, vHIT measurements both pre-surgery and post-surgery showed that the VOR gains related to ipsilesional semicircular canal stimulation are consistently impaired, while only postsurgical contralesional horizontal semicircular canal related gains were impaired relative to controls^43^. It is interesting that our DVA measures were abnormal given existing literature suggesting that measures incorporating an active head rotation afford a benefit over those involving passive head rotation (i.e. vHIT). It is well known that patients demonstrate better gaze stability for active versus passive rotations of the head on body. Specifically, compensatory saccades occur with reduced latency for active versus passive head on body rotations^17,44,45^ and DVA measures are better for active than passive head rotations^46^. In this context, electrophysiological studies in a monkey model of unilateral vestibular loss have provided insight into the mechanisms underlying the higher gaze stabilization for active versus passive head on body movements^47^. Specifically, vestibular nuclei neurons that mediate VOR show higher sensitivities to active movements due to the upweighting of motor-related inputs, while in control animals the sensitivity to active movements is comparable to the sensitivity to passive movements^23^.

One explanation for the absence of an apparent benefit during active head rotations (e.g., due to efference copy) in our present results is that we did not study the effects of rehabilitation exercises, which are known to improve DVA for active head rotations when VOR gain does not improve^29^. It is also noteworthy that we did not measure DVA during passive head rotations, nor did we measure VOR gain during active head rotations—each of which may have revealed a greater symmetry between the active and passive measures^48,49^.

Overall, we were surprised that the kinematics data revealed mean peak head velocity was comparable for patients and healthy controls even though cycle duration and range of motion were significantly greater in patients. These results suggest that although patients' head rotation could reach similar velocity as healthy controls, they require more time and distance to accelerate to the same velocity. Moreover, patients' variability of range of motion is significantly smaller than healthy controls. This further suggests that the impairment caused by a unilateral vestibular lesion leads to a reduction in the patients' ability to flexibly accelerate and decelerate their head motion, rather than a reduction in their ability to tolerate fast head motion. The kinematic data suggest that patients moved their head as fast as they could, but with a smaller acceleration compared to controls.

### Implications for the quantification of gaze stabilization exercise kinematics: patient status and real-time feedback

An exciting and unexpected finding of the present study was that correlations between pre-surgery kinematic and clinical measurements suggest that head movement cycle duration is a good indicator of patient's pre-surgical status. We found that in each of the 6 exercises, cycle duration was correlated with DVA score, mean VOR gain and standard deviations during vHIT, FGA score, completion time of TUG ipsi and contralesional trials, as well as gait speed (Fig. 5b). In addition, mean movement range and variability also provided valuable information. In contrast, measurements such as self-report questionnaires were inconsistently correlated with kinematic measurements. Our data thus indicate that these clinical measurements are more objective than those which were not correlated with kinematic measurements. Further our data suggest that specific kinematic measurements made before surgery are useful to reveal abnormal strategies recruited by patients while performing gaze stabilization exercises, which can inform exercise prescription. In particular, as shown in Fig. 6, the possibility of developing a single “kinematic score” from the three most informative kinematic measures obtained during the gaze stabilization exercises (i.e., cycle duration, mean movement range and variability) to evaluate VS patients before and after tumor resection will be an interesting direction for future work in this field.

The use of inertial measurement units (IMUs) for rehabilitative management of vestibular disorders is promising, given such technologies are evolving and will allow clinicians to both process the data offline or monitor in real-time how patients are performing their gaze stabilization exercises. In general, IMUs are becoming more commonly used to track rehabilitation outcomes^25^. Further, while traditional measures of gaze stability such as Dynamic Visual Acuity (DVA) and the video Head Impulse Test (vHIT) yield objective data that can be used to document change, their use requires specialized equipment operated by trained personnel. Overall, the approach used in the present study provides a relatively user-friendly method for measuring kinematics that can be readily adapted to quantify behavior in more ecologically relevant scenarios (i.e. walking while reading)—that can also be dangerous^50^.

Finally, our present results establish that measuring head kinematics *during* gaze stabilization exercises can be informative regarding the initial status of vestibular patients (prior to surgery) as well as the progression of their compensation following surgery. To date, numerous prior studies have provided evidence that gaze stabilization exercises benefit patients with unilateral vestibular loss. Herdman et al.^15^ first reported the utility of these exercises for unilateral patients, by showing that the performance within 3 days of VS surgery improved postural sway and disequilibrium. This finding was then furthered by Endicott et al.^51^, who reported improved Dizziness Handicap Inventory (DHI) scores for a similar patient cohort. The CPG published in 2016^13^ summarized the available clinical evidence supporting the benefits of gaze stabilization, including 5 level-1 randomized control clinical vestibular rehabilitation trials. The CPG concluded that early initiation of customized, supervised, gaze stability training reduced fall risk and dizziness complaints, thereby improving health related quality of life. Most recently, studies have also reported specific improvements in active DVA, passive VOR gain, as well as compensatory saccades, and subjective dizziness (DHI) in acute patients^48,52^. After 5 weeks of vestibular rehabilitation, improvements were observed in many subjective and functional measures, including DVA but not passive VOR gains^29^. Importantly, however, all prior studies to date focused on the benefits of gaze stabilization exercises without real-time feedback about the performance. Previous motor learning studies have shown that real-time feedback (in contrast to delayed feedback) can greatly benefit motor adaptation^53–55^. Thus, we expect that providing patients with real-time feedback of kinematic measures, such as those quantified in the present study as well as other recent investigations^56,57^, will lead to greater improvements in the performance of unilateral vestibular loss patients.

## Supplementary Information

Supplementary Information.
