# Supplementary material for: Head movement kinematics are altered during gaze stability exercises in vestibular schwannoma patients
Source: Sci Rep. 2021 Mar 30;11:7139. doi: 10.1038/s41598-021-86533-3 (PMC8010068; doi:10.1038/s41598-021-86533-3)
Supplement: Supplementary file 1 — Supplementary Information. [file 41598_2021_86533_MOESM1_ESM.docx]

**Head movement kinematics are altered during gaze stability exercises in vestibular schwannoma patients**

Lin Wang^1#^, Omid A. Zobeiri^2#^, Jennifer L. Millar^3^, Michael C. Schubert^3,4^, Kathleen E. Cullen^1,4,5,6*^

^1^ Department of Biomedical Engineering, Johns Hopkins University, Baltimore, USA.

^2^ Department of Biomedical Engineering, McGill University, Montreal, QC, Canada

^3^ Department of Physical Medicine and Rehabilitation, Johns Hopkins University School of

Medicine, Baltimore, USA.

^4^ Department of Otolaryngology-Head and Neck Surgery, Johns Hopkins University School of

Medicine, Baltimore, USA.

^5^ Department of Neuroscience, Johns Hopkins University School of Medicine, Baltimore, USA.

^6^ Kavli Neuroscience Discovery Institute, Johns Hopkins University, Baltimore, USA.

*Correspondence: [kathleen.cullen@jhu.edu](mailto:kathleen.cullen@jhu.edu)

#These authors also contributed equally to this work

| Supplementary Table 1  Correlation coefficients (slope) for Task 1 (Preop. Clinical vs. Preop. Kinematics) | | | | | | | | | | | | | | | | |
| --- | --- | --- | --- | --- | --- | --- | --- | --- | --- | --- | --- | --- | --- | --- | --- | --- |
| Clinical Measures | Functional |  | DVA | IPSI | -0.43 | 0.66 | 0.49 | 0.43 | -0.051 | -0.47 | 0.63 | -0.4 | -0.11 | -0.15 | -0.24 | 0.17 |
|  |  |  |  | CONTRA | -4.70E-01 | 0.74 (3.9) | 0.45 | 0.4 | 0.011 | -0.51 | 0.72 (0.58) | -0.47 | -0.21 | 0.056 | -0.27 | 0.33 |
|  |  |  | TUG | IPSI | -0.39 | 0.55 | 0.35 | 0.73 (0.014) | 0.081 | -0.34 | 0.7 | -0.58 | 0.32 | -0.32 | -0.55 | 0.21 |
|  |  |  |  | CONTRA | -4.50E-01 | 0.53 | 0.29 | 0.74 (0.015) | 0.15 | -0.26 | 0.7 | -0.54 | 0.35 | -0.29 | -0.51 | 0.19 |
|  |  |  |  | GAIT SPEED | 0.63 | -0.58 | -0.059 | -0.35 | -0.075 | 0.13 | -0.48 | 0.13 | 0.094 | -0.24 | 0.0068 | -0.23 |
|  |  |  |  | FGA | 0.53 | -0.49 | -0.19 | -0.23 | -0.064 | 0.17 | -0.45 | 0.22 | 0.43 | -0.077 | -0.47 | -0.0076 |
|  | Physiological (VHIT) | IPSILESIONAL | MEAN | Horizontal | 0.78 (260) | -0.81 (-1.8) | -0.35 | -0.011 | -0.34 | 0.36 | -0.6 | 0.35 | 0.079 | -0.13 | 0.24 | -0.73 (-1.3) |
|  |  |  |  | Posterior | 0.22 | -0.53 | -0.31 | -0.084 | 0.68 | 0.57 | -0.11 | 0.14 | 0.47 | -0.072 | -0.22 | -0.44 |
|  |  |  |  | Anterior | 0.35 | -0.32 | -0.29 | -0.57 | -0.74 (-0.32) | 0.066 | -0.82 (-0.3) | 0.87 (0.18) | -0.32 | 0.43 | 0.32 | -0.11 |
|  |  |  | STANDARD  DEVIATION | Horizontal | -0.36 | 0.53 | 0.41 | 0.69 | 0.31 | -0.32 | 0.82 (0.83) | -0.65 | 0.27 | -0.3 | -0.58 | 0.11 |
|  |  |  |  | Posterior | 0.43 | -0.093 | -0.072 | 0.3 | -0.56 | -0.21 | -0.044 | -0.13 | -0.2 | 0.18 | -0.032 | -0.04 |
|  |  |  |  | Anterior | -0.48 | 0.5 | 0.33 | 0.54 | 0.31 | -0.19 | 0.68 | -0.42 | 0.17 | -0.24 | -0.31 | 0.0012 |
|  |  | CONTRALESIONAL | MEAN | Horizontal | 0.49 | 0.047 | 0.4 | 0.16 | -0.35 | -0.48 | 0.22 | -0.32 | -0.39 | -0.03 | -0.18 | -0.2 |
|  |  |  |  | Posterior | 0.57 | -0.62 | -0.32 | -0.3 | -0.27 | 0.29 | -0.56 | 0.44 | -0.35 | 0.19 | 0.59 | -0.6 |
|  |  |  |  | Anterior | 0.034 | -0.4 | -0.49 | -0.0065 | 0.37 | 0.59 | -0.14 | 0.37 | 0.045 | 0.32 | 0.3 | -0.55 |
|  |  |  | STANDARD  DEVIATION | Horizontal | 0.14 | -0.25 | -0.093 | 0.14 | 0.52 | 0.27 | 0.24 | -0.27 | -0.14 | -0.015 | 0.31 | -0.48 |
|  |  |  |  | Posterior | -0.18 | 0.36 | 0.15 | 0.76 (0.5) | 0.047 | -0.22 | 0.62 | -0.49 | 0.27 | -0.087 | -0.54 | 0.078 |
|  |  |  |  | Anterior | -0.16 | 0.14 | -0.13 | 0.3 | 0.36 | 0.069 | 0.47 | -0.47 | -0.32 | 0.3 | 0.33 | 0.012 |
|  | Subjective |  |  | DHI | -0.1 | 0.37 | 0.41 | 0.37 | -0.023 | -0.4 | 0.57 | -0.6 | -0.47 | -0.14 | 0.26 | -0.069 |
|  |  |  |  | ABC | -0.17 | 0.044 | -0.11 | -0.24 | 0.17 | 0.11 | -0.14 | 0.29 | 0.4 | 0.15 | -0.5 | 0.33 |
|  |  |  |  | HEADACHE | 0.07 | -0.081 | -0.011 | 0.71 (0.005) | 0.23 | 0.13 | 0.42 | -0.31 | 0.32 | -0.25 | -0.26 | -0.52 |
|  |  |  |  | BECK ANXIETY | 0.11 | -0.0039 | 0.3 | 0.66 | 0.49 | -0.075 | 0.63 | -0.85 (-0.008) | 0.37 | -0.65 | -0.36 | -0.23 |
|  |  |  |  |  | Peak  Vel. | Cycle  Dur. | Mov.  range | Peak  Vel. | Cycle  Dur. | Mov.  range | Peak  Vel. | Cycle  Dur. | Mov.  range | Peak  Vel. | Cycle  Dur. | Mov.  range |
|  |  |  |  |  | Mean | | | CV | | | Mean | | | CV | | |
|  |  |  |  |  |  | | | | | | Asymmetry | | | | | |
|  |  |  |  |  | Kinematic Measures | | | | | | | | | | | |

| Supplementary Table 2  Correlation coefficients (slope) for Task 2 (Preop. Clinical vs. Preop. Kinematics) | | | | | | | | | | | | | | | | |
| --- | --- | --- | --- | --- | --- | --- | --- | --- | --- | --- | --- | --- | --- | --- | --- | --- |
| Clinical Measures | Functional |  | DVA | IPSI | -0.23 | 0.81 (2.3) | 0.76 (35) | -0.34 | -0.037 | -0.65 | 0.26 | -0.16 | 0.63 | -0.014 | 0.46 | -0.77 (-1.3) |
|  |  |  |  | CONTRA | -0.24 | 0.87 (4.2) | 0.79 (63) | -0.36 | 0.063 | -0.64 | 0.088 | 0.15 | 0.69 | -0.061 | 0.26 | -0.82 (-2.4) |
|  |  |  | TUG | IPSI | -0.41 | 0.72 (0.14) | 0.58 | -0.085 | -0.19 | -0.33 | 0.56 | -0.29 | 0.64 | -0.11 | -0.015 | -0.64 |
|  |  |  |  | CONTRA | -4.60E-01 | 0.71 (0.14) | 0.54 | -0.1 | -0.12 | -0.29 | 0.55 | -0.29 | 0.65 | -0.18 | 0.0081 | -0.62 |
|  |  |  |  | GAIT SPEED | 0.37 | -0.7 | -0.51 | 0.41 | -0.093 | 0.44 | -0.085 | -0.0054 | -0.5 | 0.29 | -0.39 | 0.49 |
|  |  |  |  | FGA | 0.26 | -0.52 | -0.35 | 0.15 | -0.23 | 0.5 | 0.27 | -0.12 | -0.12 | -0.11 | -0.68 | 0.54 |
|  | Physiological (VHIT) | IPSILESIONAL | MEAN | Horizontal | 0.71 (150) | -0.79 (-1.6) | -0.57 | 0.72 (0.14) | -0.088 | 0.53 | -0.41 | 0.019 | -0.74 (-0.3) | 0.68 | 0.17 | 0.39 |
|  |  |  |  | Posterior | 0.051 | -0.48 | -0.44 | 0.086 | 0.75 (0.24) | 0.58 | -0.012 | 0.15 | 0.23 | -0.48 | -0.24 | 0.23 |
|  |  |  |  | Anterior | 0.6 | -0.34 | -0.081 | -0.23 | -0.44 | -0.053 | -0.24 | -0.14 | -0.47 | 0.24 | 0.44 | 0.52 |
|  |  |  | STANDARD  DEVIATION | Horizontal | -0.35 | 0.72 (4.4) | 0.62 | -0.12 | 0.18 | -0.33 | 0.39 | -0.061 | 0.82 (0.99) | -0.18 | 0.027 | -0.8 (-2.9) |
|  |  |  |  | Posterior | 0.48 | -0.054 | 0.024 | 0.55 | -0.42 | 0.073 | -0.43 | 0.39 | -0.41 | 0.77 (2.5) | -0.15 | -0.12 |
|  |  |  |  | Anterior | -0.37 | 0.68 | 0.57 | -0.27 | 0.27 | -0.39 | 0.34 | -0.18 | 0.75 (1.4) | -0.27 | 0.36 | -0.71 (-4.1) |
|  |  | CONTRALESIONAL | MEAN | Horizontal | 0.65 | 0.13 | 0.4 | 0.3 | 0.055 | -0.26 | -0.5 | 0.52 | 0.068 | 0.74 (0.9) | 0.18 | -0.59 |
|  |  |  |  | Posterior | 6.90E-01 | -0.67 | -0.47 | 0.46 | 0.18 | 0.3 | -0.77 (-0.33) | 0.35 | -0.73 (-0.48) | 0.61 | 0.4 | 0.3 |
|  |  |  |  | Anterior | 0.19 | -0.32 | -0.33 | 0.074 | 0.73 (0.26) | 0.35 | -0.49 | 0.35 | -0.014 | -0.19 | 0.39 | 0.074 |
|  |  |  | STANDARD  DEVIATION | Horizontal | 0.14 | -0.26 | -0.3 | 0.46 | 0.82 (4.2) | 0.27 | -0.66 | 0.63 | -0.1 | 0.2 | 0.11 | -0.26 |
|  |  |  |  | Posterior | -0.15 | 0.56 | 0.46 | 0.11 | -0.049 | -0.14 | 0.21 | 0.063 | 0.53 | 0.036 | -0.089 | -0.62 |
|  |  |  |  | Anterior | -0.11 | 0.091 | -0.15 | 0.44 | 0.52 | 0.12 | -0.73 (-1.8) | 0.87 (1.7) | -0.13 | 0.23 | -0.19 | -0.36 |
|  | Subjective |  |  | DHI | 0.031 | 0.39 | 0.32 | 0.32 | 0.17 | -0.39 | -0.45 | 0.4 | -0.016 | 0.59 | 0.34 | -0.72 (-0.011) |
|  |  |  |  | ABC | -0.24 | 0.072 | 0.084 | -0.58 | -0.051 | 0.072 | 0.56 | -0.28 | 0.49 | -0.77 (-0.009) | -0.38 | 0.31 |
|  |  |  |  | HEADACHE | 0.1 | 0.14 | 0.13 | 0.37 | 0.37 | 0.11 | -0.053 | 0.074 | 0.32 | 0.18 | 0.29 | -0.53 |
|  |  |  |  | BECK ANXIETY | -0.2 | 0.05 | -0.049 | 0.63 | 0.28 | 0.21 | 0.14 | 0.091 | 0.19 | 0.23 | -0.38 | -0.48 |
|  |  |  |  |  | Peak  Vel. | Cycle  Dur. | Mov.  range | Peak  Vel. | Cycle  Dur. | Mov.  range | Peak  Vel. | Cycle  Dur. | Mov.  range | Peak  Vel. | Cycle  Dur. | Mov.  range |
|  |  |  |  |  | Mean | | | CV | | | Mean | | | CV | | |
|  |  |  |  |  |  | | | | | | Asymmetry | | | | | |
|  |  |  |  |  | Kinematic Measures | | | | | | | | | | | |

| Supplementary Table 3  Correlation coefficients (slope) for Task 3 (Preop. Clinical vs. Preop. Kinematics) | | | | | | | | | | | | | | | | |
| --- | --- | --- | --- | --- | --- | --- | --- | --- | --- | --- | --- | --- | --- | --- | --- | --- |
| Clinical Measures | Functional |  | DVA | IPSI | -0.42 | 0.86 (2.5) | 0.56 | 0.16 | 0.89 (0.18) | -0.36 | 0.55 | -0.64 | 0.26 | 0.37 | 0.081 | -0.55 |
|  |  |  |  | CONTRA | -3.90E-01 | 0.89 (4.4) | 0.55 | -0.036 | 0.77 (0.27) | -0.59 | 0.61 | -0.65 | 0.47 | 0.49 | 0.2 | -0.55 |
|  |  |  | TUG | IPSI | -0.48 | 0.75 (0.14) | 0.38 | 0.44 | 0.74 (0.01) | -0.16 | 0.71 (0.021) | -0.57 | 0.31 | 0.4 | -0.044 | -0.32 |
|  |  |  |  | CONTRA | -5.20E-01 | 0.73 (0.15) | 0.31 | 0.48 | 0.78 (0.011) | -0.14 | 0.66 | -0.54 | 0.3 | 0.45 | -0.079 | -0.28 |
|  |  |  |  | GAIT SPEED | 0.49 | -0.69 | -0.16 | -0.27 | -0.8 (-0.11) | 0.32 | -0.25 | 0.42 | -0.15 | -0.41 | -0.12 | 0.24 |
|  |  |  |  | FGA | 0.27 | -0.53 | -0.21 | -0.036 | -0.74 (-0.008) | 0.46 | -0.15 | 0.54 | -0.13 | -0.45 | -0.26 | 0.64 |
|  | Physiological (VHIT) | IPSILESIONAL | MEAN | Horizontal | 0.89 (240) | -0.75 (-1.5) | -0.3 | 0.2 | -0.41 | 0.37 | -0.13 | -0.14 | -0.6 | -0.27 | -0.37 | -0.0072 |
|  |  |  |  | Posterior | 0.13 | -0.47 | -0.36 | -0.12 | -0.12 | 0.22 | -0.15 | 0.46 | 0.22 | 0.31 | -0.67 | 0.36 |
|  |  |  |  | Anterior | 0.41 | -0.32 | -0.13 | 0.066 | -0.31 | 0.37 | -0.56 | 0.2 | -0.83 (-0.23) | -0.75 (-0.72) | 0.065 | 0.35 |
|  |  |  | STANDARD  DEVIATION | Horizontal | -0.42 | 0.76 (4.7) | 0.44 | 0.2 | 0.82 (0.36) | -0.35 | 0.78 (0.77) | -0.6 | 0.55 | 0.64 | -0.17 | -0.45 |
|  |  |  |  | Posterior | 0.63 | -0.085 | 0.077 | 0.069 | -0.28 | -0.28 | 0.4 | -0.64 | -0.14 | -0.076 | 0.22 | -0.28 |
|  |  |  |  | Anterior | -0.49 | 0.73 (7.3) | 0.36 | 0.26 | 0.97 (0.68) | -0.24 | 0.52 | -0.5 | 0.36 | 0.59 | -0.2 | -0.42 |
|  |  | CONTRALESIONAL | MEAN | Horizontal | 0.65 | 0.19 | 0.54 | -0.4 | 0.053 | -0.57 | 0.62 | -0.78 (-0.26) | 0.25 | 0.21 | 0.014 | -0.71 (-1.3) |
|  |  |  |  | Posterior | 0.83 (380) | -0.64 | -0.25 | -0.17 | -0.37 | 0.03 | -0.32 | -0.1 | -0.45 | -0.16 | -0.12 | -0.15 |
|  |  |  |  | Anterior | 0.27 | -0.31 | -0.4 | 0.06 | 0.25 | -0.014 | -0.21 | -0.0065 | -0.091 | 0.41 | -0.47 | 0.047 |
|  |  |  | STANDARD  DEVIATION | Horizontal | 0.39 | -0.25 | -0.12 | -0.35 | 0.058 | -0.44 | 0.15 | -0.26 | 0.42 | 0.66 | -0.23 | -0.47 |
|  |  |  |  | Posterior | -0.14 | 0.56 | 0.26 | 0.37 | 0.59 | -0.3 | 0.77 (0.78) | -0.71 | 0.32 | 0.5 | -0.12 | -0.32 |
|  |  |  |  | Anterior | 0.2 | 0.0045 | -0.12 | -0.33 | -0.02 | -0.76 (-3.9) | 0.31 | -0.44 | 0.58 | 0.7 | 0.26 | -0.49 |
|  | Subjective |  |  | DHI | 0.15 | 0.41 | 0.42 | -0.23 | 0.4 | -0.7 | 0.54 | -0.82 (-0.002) | 0.39 | 0.52 | 0.29 | -0.93 (-0.014) |
|  |  |  |  | ABC | -0.44 | 0.046 | -0.11 | 0.06 | -0.096 | 0.36 | -0.24 | 0.64 | 0.026 | -0.23 | -0.17 | 0.72 (0.013) |
|  |  |  |  | HEADACHE | 0.19 | 0.2 | 0.083 | 0.39 | 0.68 | -0.14 | 0.61 | -0.71 (-0.005) | 0.14 | 0.64 | -0.52 | -0.45 |
|  |  |  |  | BECK ANXIETY | 0.047 | 0.077 | 0.17 | 0.021 | 0.15 | -0.23 | 0.69 | -0.42 | 0.6 | 0.61 | -0.24 | -0.49 |
|  |  |  |  |  | Peak  Vel. | Cycle  Dur. | Mov.  range | Peak  Vel. | Cycle  Dur. | Mov.  range | Peak  Vel. | Cycle  Dur. | Mov.  range | Peak  Vel. | Cycle  Dur. | Mov.  range |
|  |  |  |  |  | Mean | | | CV | | | Mean | | | CV | | |
|  |  |  |  |  |  | | | | | | Asymmetry | | | | | |
|  |  |  |  |  | Kinematic Measures | | | | | | | | | | | |

| Supplementary Table 4  Correlation coefficients (slope) for Task 4 (Preop. Clinical vs. Preop. Kinematics) | | | | | | | | | | | | | | | | |
| --- | --- | --- | --- | --- | --- | --- | --- | --- | --- | --- | --- | --- | --- | --- | --- | --- |
| Clinical Measures | Functional |  | DVA | IPSI | -0.23 | 0.85 (2.3) | 0.54 | -0.13 | 0.0079 | -0.55 | 0.64 | -0.55 | 0.48 | -0.88 (-0.93) | -0.17 | -0.014 |
|  |  |  |  | CONTRA | -0.22 | 0.89 (4.1) | 0.56 | -0.13 | 0.17 | -0.53 | 0.48 | -0.4 | 0.48 | -0.82 (-1.5) | 0.027 | 0.16 |
|  |  |  | TUG | IPSI | -0.47 | 0.79 (0.14) | 0.34 | 0.077 | -0.16 | -0.32 | 0.73 (0.025) | -0.74 (-0.027) | 0.41 | -0.53 | -0.28 | 0.21 |
|  |  |  |  | CONTRA | -0.5 | 0.78 (0.15) | 0.28 | 0.074 | -0.083 | -0.26 | 0.71 (0.026) | -0.73 (-0.028) | 0.43 | -0.55 | -0.3 | 0.24 |
|  |  |  |  | GAIT SPEED | 0.28 | -0.7 | -0.31 | 0.089 | -0.26 | 0.26 | -0.4 | 0.34 | -0.36 | 0.76 (0.53) | -0.079 | -0.25 |
|  |  |  |  | FGA | 0.26 | -0.46 | -0.1 | -0.16 | -0.22 | 0.22 | -0.016 | -0.075 | -0.11 | 0.83 (0.044) | 0.13 | 0.45 |
|  | Physiological (VHIT) | IPSILESIONAL | MEAN | Horizontal | 0.59 | -0.78 (-1.5) | -0.48 | 0.55 | -0.16 | 0.42 | -0.37 | 0.39 | -0.69 | 0.4 | -0.011 | -0.53 |
|  |  |  |  | Posterior | 0.092 | -0.44 | -0.34 | -0.23 | 0.73 (0.24) | 0.48 | -0.099 | 0.019 | 0.4 | 0.21 | -0.3 | 0.4 |
|  |  |  |  | Anterior | 0.74 (150) | -0.34 | 0.17 | -0.19 | -0.26 | -0.14 | -0.073 | 0.2 | -0.42 | 0.13 | 0.6 | -0.021 |
|  |  |  | STANDARD  DEVIATION | Horizontal | -0.4 | 0.79 (4.7) | 0.36 | -0.032 | 0.19 | -0.31 | 0.66 | -0.66 | 0.62 | -0.67 | -0.35 | 0.25 |
|  |  |  |  | Posterior | 0.35 | -0.072 | -0.056 | 0.63 | -0.34 | 0.02 | -0.24 | 0.29 | -0.69 | 0.21 | 0.44 | -0.23 |
|  |  |  |  | Anterior | -0.36 | 0.73 (6.9) | 0.33 | -0.13 | 0.29 | -0.3 | 0.65 | -0.62 | 0.65 | -0.85 (-3.1) | -0.39 | 0.13 |
|  |  | CONTRALESIONAL | MEAN | Horizontal | 0.52 | 0.15 | 0.3 | 0.23 | 0.061 | -0.33 | -0.054 | 0.17 | -0.12 | -0.3 | 0.16 | -0.34 |
|  |  |  |  | Posterior | 0.64 | -0.72 (-2.3) | -0.39 | 0.37 | 0.15 | 0.31 | -0.63 | 0.71 (0.46) | -0.61 | 0.16 | 0.18 | -0.59 |
|  |  |  |  | Anterior | 0.27 | -0.32 | -0.34 | 0.059 | 0.84 (0.31) | 0.43 | -0.25 | 0.28 | 0.089 | -0.28 | 0.0008 | 0.1 |
|  |  |  | STANDARD  DEVIATION | Horizontal | 0.032 | -0.31 | -0.44 | 0.36 | 0.7 | 0.39 | -0.55 | 0.55 | -0.022 | -0.16 | -0.28 | -0.39 |
|  |  |  |  | Posterior | -0.22 | 0.62 | 0.22 | 0.25 | 0.057 | -0.15 | 0.5 | -0.5 | 0.22 | -0.43 | -0.049 | 0.29 |
|  |  |  |  | Anterior | -0.22 | -0.013 | -0.38 | 0.55 | 0.51 | 0.33 | -0.67 | 0.65 | -0.25 | -0.073 | 0.093 | -0.22 |
|  | Subjective |  |  | DHI | -0.13 | 0.32 | 0.037 | 0.46 | 0.05 | -0.2 | -0.19 | 0.26 | -0.13 | -0.54 | -0.18 | -0.7 |
|  |  |  |  | ABC | -0.56 | 0.14 | 0.28 | -0.67 | 0.1 | -0.076 | 0.42 | -0.47 | 0.51 | 0.21 | 0.16 | 0.9 (0.014) |
|  |  |  |  | HEADACHE | 0.0071 | 0.21 | -0.1 | 0.38 | 0.38 | 0.13 | 0.32 | -0.29 | 0.18 | -0.54 | -0.35 | -0.074 |
|  |  |  |  | BECK ANXIETY | -0.45 | 0.075 | -0.29 | 0.48 | 0.036 | 0.2 | 0.025 | -0.13 | 0.11 | 0.025 | -0.67 | -0.31 |
|  |  |  |  |  | Peak  Vel. | Cycle  Dur. | Mov.  range | Peak  Vel. | Cycle  Dur. | Mov.  range | Peak  Vel. | Cycle  Dur. | Mov.  range | Peak  Vel. | Cycle  Dur. | Mov.  range |
|  |  |  |  |  | Mean | | | CV | | | Mean | | | CV | | |
|  |  |  |  |  |  | | | | | | Asymmetry | | | | | |
|  |  |  |  |  | Kinematic Measures | | | | | | | | | | | |

| Supplementary Table 5  Correlation coefficients (slope) for Task 5 (Preop. Clinical vs. Preop. Kinematics) | | | | | | | | | | | | | | | | |
| --- | --- | --- | --- | --- | --- | --- | --- | --- | --- | --- | --- | --- | --- | --- | --- | --- |
| Clinical Measures | Functional |  | DVA | IPSI | -0.35 | 0.86 (2.5) | 0.64 | -0.48 | 0.96 (0.36) | -0.57 | 0.83 (0.39) | -0.58 | 0.32 | -0.039 | -0.0078 | -0.45 |
|  |  |  |  | CONTRA | -0.32 | 0.89 (4.5) | 0.66 | -0.53 | 0.92 (0.6) | -0.63 | 0.92 (0.75) | -0.67 | 0.45 | -0.26 | 0.16 | -0.44 |
|  |  |  | TUG | IPSI | -0.5 | 0.7 | 0.36 | -0.017 | 0.72 (0.018) | -0.32 | 0.78 (0.024) | -0.87 (-0.027) | 0.16 | 0.16 | -0.016 | -0.47 |
|  |  |  |  | CONTRA | -0.53 | 0.69 | 0.31 | -0.022 | 0.74 (0.02) | -0.28 | 0.77 (0.025) | -0.85 (-0.028) | 0.08 | 0.13 | -0.062 | -0.51 |
|  |  |  |  | GAIT SPEED | 0.43 | -0.71 (-1.4) | -0.29 | 0.57 | -0.84 (-0.21) | 0.3 | -0.68 | 0.47 | -0.084 | 0.27 | 0.0047 | 0.38 |
|  |  |  |  | FGA | 0.11 | -0.59 | -0.43 | 0.59 | -0.76 (-0.015) | 0.22 | -0.52 | 0.01 | -0.22 | 0.42 | 0.074 | 0.38 |
|  | Physiological (VHIT) | IPSILESIONAL | MEAN | Horizontal | 0.87 (280 | -0.76 (-1.6) | -0.26 | 0.31 | -0.5 | 0.5 | -0.64 | 0.63 | 0.033 | 0.33 | -0.55 | 0.02 |
|  |  |  |  | Posterior | 0.054 | -0.46 | -0.31 | 0.44 | -0.26 | 0.23 | -0.19 | 0.16 | -0.54 | 0.21 | -0.46 | -0.39 |
|  |  |  |  | Anterior | 0.39 | -0.32 | -0.17 | -0.47 | -0.23 | 0.062 | -0.55 | 0.55 | 0.057 | 0.11 | -0.033 | 0.57 |
|  |  |  | STANDARD  DEVIATION | Horizontal | -0.41 | 0.73 (4.6) | 0.5 | -0.056 | 0.83 (0.68) | -0.45 | 0.91 (0.93) | -0.84 (-0.87) | 0.19 | 0.064 | -0.11 | -0.7 |
|  |  |  |  | Posterior | 0.64 | -0.11 | 0.11 | -0.021 | -0.13 | 0.077 | 0.012 | -0.071 | 0.66 | -0.14 | 0.077 | 0.066 |
|  |  |  |  | Anterior | -0.45 | 0.73 (7.4) | 0.46 | -0.25 | 0.93 (1.2) | -0.41 | 0.82 (1.3) | -0.6 | 0.02 | 0.05 | -0.24 | -0.69 |
|  |  | CONTRALESIONAL | MEAN | Horizontal | 0.7 | 0.18 | 0.66 | -0.23 | 0.3 | -0.45 | 0.37 | -0.07 | 0.83 (0.2) | -0.1 | -0.061 | -0.31 |
|  |  |  |  | Posterior | 0.89 (480) | -0.6 | -0.11 | -0.092 | -0.36 | 0.33 | -0.55 | 0.84 (0.47) | 0.15 | -0.085 | -0.38 | 0.058 |
|  |  |  |  | Anterior | 0.3 | -0.25 | -0.17 | -0.17 | 0.16 | 0.25 | -0.053 | 0.37 | -0.29 | -0.14 | -0.61 | -0.5 |
|  |  |  | STANDARD  DEVIATION | Horizontal | 0.49 | -0.2 | 0.13 | 0.12 | 0.038 | 0.14 | 0.096 | 0.33 | 0.076 | -0.32 | -0.38 | -0.58 |
|  |  |  |  | Posterior | -0.16 | 0.53 | 0.31 | -0.038 | 0.63 | -0.24 | 0.74 (0.78) | -0.79 (-0.84) | 0.3 | 0.025 | -0.11 | -0.56 |
|  |  |  |  | Anterior | 0.32 | 0.061 | 0.12 | -0.028 | 0.067 | 0.11 | 0.31 | -0.0019 | 0.35 | -0.71 (-3.6) | 0.082 | -0.39 |
|  | Subjective |  |  | DHI | 0.31 | 0.44 | 0.62 | -0.26 | 0.51 | -0.27 | 0.53 | -0.066 | 0.63 | -0.39 | 0.0079 | -0.42 |
|  |  |  |  | ABC | -0.57 | 0.019 | -0.28 | 0.077 | -0.12 | -0.11 | -0.039 | -0.33 | -0.44 | 0.24 | 0.18 | 0.22 |
|  |  |  |  | HEADACHE | 0.21 | 0.19 | 0.26 | 0.056 | 0.6 | -0.014 | 0.51 | -0.31 | 0.095 | 0.17 | -0.65 | -0.86 (-0.047) |
|  |  |  |  | BECK ANXIETY | 0.058 | 0.042 | 0.2 | 0.66 | 0.058 | 0.056 | 0.35 | -0.37 | 0.11 | 0.14 | -0.2 | -0.58 |
|  |  |  |  |  | Peak  Vel. | Cycle  Dur. | Mov.  range | Peak  Vel. | Cycle  Dur. | Mov.  range | Peak  Vel. | Cycle  Dur. | Mov.  range | Peak  Vel. | Cycle  Dur. | Mov.  range |
|  |  |  |  |  | Mean | | | CV | | | Mean | | | CV | | |
|  |  |  |  |  |  | | | | | | Asymmetry | | | | | |
|  |  |  |  |  | Kinematic Measures | | | | | | | | | | | |

| Supplementary Table 6  Correlation coefficients (slope) for Task 6 (Preop. Clinical vs. Preop. Kinematics) | | | | | | | | | | | | | | | | |
| --- | --- | --- | --- | --- | --- | --- | --- | --- | --- | --- | --- | --- | --- | --- | --- | --- |
| Clinical Measures | Functional |  | DVA | IPSI | -0.035 | 0.84 (2.2) | 0.81 (35) | -0.38 | 0.14 | -0.2 | -0.0013 | -0.19 | 0.36 | -0.41 | 0.32 | 0.18 |
|  |  |  |  | CONTRA | -0.098 | 0.89 (3.9) | 0.87 (65) | -0.5 | 0.13 | -0.28 | -0.079 | -0.16 | 0.38 | -0.29 | 0.37 | 0.32 |
|  |  |  | TUG | IPSI | -0.35 | 0.74 (0.13) | 0.59 | -0.025 | -0.31 | 0.15 | -0.26 | -0.098 | 0.45 | -0.15 | 0.08 | 0.44 |
|  |  |  |  | CONTRA | -0.37 | 0.73 (0.13) | 0.56 | -0.069 | -0.24 | 0.2 | -0.27 | -0.072 | 0.43 | -0.1 | 0.096 | 0.46 |
|  |  |  |  | GAIT SPEED | 0.049 | -0.74 (-1.3) | -0.68 | 0.62 | -0.29 | 0.15 | 0.21 | -0.0045 | -0.19 | 0.039 | -0.49 | -0.39 |
|  |  |  |  | FGA | -0.065 | -0.55 | -0.43 | 0.32 | -0.59 | 0.22 | 0.066 | -0.26 | 0.32 | 0.29 | -0.3 | 0.19 |
|  | Physiological (VHIT) | IPSILESIONAL | MEAN | Horizontal | 0.64 | -0.78 (-1.4) | -0.47 | 0.45 | -0.12 | 0.15 | -0.27 | 0.51 | -0.43 | -0.35 | -0.69 | -0.77 (-1) |
|  |  |  |  | Posterior | -0.078 | -0.49 | -0.46 | -0.1 | 0.33 | 0.56 | 0.24 | -0.13 | 0.27 | 0.25 | -0.36 | 0.016 |
|  |  |  |  | Anterior | 0.7 | -0.3 | 0.12 | -0.37 | 0.02 | -0.51 | 0.03 | -0.057 | -0.079 | -0.17 | 0.28 | -0.24 |
|  |  |  | STANDARD  DEVIATION | Horizontal | -0.27 | 0.73 (4.1) | 0.62 | -0.19 | -0.045 | 0.18 | -0.11 | -0.16 | 0.53 | -0.22 | 0.023 | 0.37 |
|  |  |  |  | Posterior | 0.55 | -0.06 | 0.19 | 0.24 | -0.52 | -0.26 | -0.65 | 0.51 | -0.26 | -0.29 | -0.32 | -0.23 |
|  |  |  |  | Anterior | -0.21 | 0.71 (6.3) | 0.6 | -0.36 | 0.26 | 0.14 | 0.01 | -0.16 | 0.42 | -0.25 | 0.17 | 0.25 |
|  |  | CONTRALESIONAL | MEAN | Horizontal | 0.7 | 0.12 | 0.48 | -0.062 | -0.044 | -0.36 | -0.11 | 0.12 | 0.084 | -0.71 (-1) | -0.32 | -0.46 |
|  |  |  |  | Posterior | 0.76 (280) | -0.66 | -0.35 | 0.08 | 0.34 | -0.14 | -0.09 | 0.49 | -0.59 | -0.34 | -0.38 | -0.82 (-1.8) |
|  |  |  |  | Anterior | 0.41 | -0.28 | -0.11 | -0.5 | 0.7 | 0.22 | -0.1 | 0.3 | -0.13 | -0.0035 | -0.12 | -0.23 |
|  |  |  | STANDARD  DEVIATION | Horizontal | 0.21 | -0.28 | -0.29 | 0.063 | 0.58 | 0.26 | -0.039 | 0.45 | -0.4 | -0.16 | -0.47 | -0.51 |
|  |  |  |  | Posterior | -0.02 | 0.58 | 0.59 | -0.14 | -0.3 | 0.12 | -0.49 | 0.12 | 0.38 | -0.18 | -0.072 | 0.35 |
|  |  |  |  | Anterior | 0.073 | 0.057 | -0.068 | 0.038 | 0.28 | 0.034 | -0.42 | 0.63 | -0.52 | 0.086 | -0.21 | -0.13 |
|  | Subjective |  |  | DHI | 0.19 | 0.37 | 0.32 | 0.14 | 0.24 | -0.2 | -0.17 | 0.4 | -0.39 | -0.51 | -0.16 | -0.45 |
|  |  |  |  | ABC | -0.35 | 0.077 | 0.068 | -0.38 | -0.17 | 0.1 | 0.26 | -0.62 | 0.68 | 0.48 | 0.37 | 0.72 (0.015) |
|  |  |  |  | HEADACHE | 0.25 | 0.18 | 0.28 | -0.081 | 0.14 | 0.36 | -0.37 | 0.33 | 0.15 | -0.41 | -0.42 | -0.18 |
|  |  |  |  | BECK ANXIETY | -0.39 | 0.0068 | -0.24 | 0.7 | -0.23 | 0.55 | -0.15 | 0.24 | -0.034 | -0.15 | -0.68 | -0.17 |
|  |  |  |  |  | Peak  Vel. | Cycle  Dur. | Mov.  range | Peak  Vel. | Cycle  Dur. | Mov.  range | Peak  Vel. | Cycle  Dur. | Mov.  range | Peak  Vel. | Cycle  Dur. | Mov.  range |
|  |  |  |  |  | Mean | | | CV | | | Mean | | | CV | | |
|  |  |  |  |  |  | | | | | | Asymmetry | | | | | |
|  |  |  |  |  | Kinematic Measures | | | | | | | | | | | |

| Supplementary Table 7  Correlation coefficients (slope) for Task 1 (Postop. Clinical vs. Postop. Kinematics) | | | | | | | | | | | | | | | | |
| --- | --- | --- | --- | --- | --- | --- | --- | --- | --- | --- | --- | --- | --- | --- | --- | --- |
| Clinical Measures | Functional |  | DVA | IPSI | -0.86 (-270) | 0.75 | 0.28 | 0.14 | 0.34 | -0.22 | -0.24 | -0.31 | 0.44 | -0.37 | 0.71 | 0.23 |
|  |  |  |  | CONTRA | -0.81 (-280) | 0.85 (3.1) | 0.69 | -0.082 | 0.36 | -0.66 | -0.44 | -0.055 | -0.16 | 0.23 | 0.22 | -0.38 |
|  |  |  | TUG | IPSI | -0.54 | 0.87 (0.077) | 0.59 | 0.31 | 0.35 | -0.052 | -0.46 | -0.54 | 0.53 | -0.53 | 0.74 | -0.091 |
|  |  |  |  | CONTRA | -0.57 | 0.86 (0.085) | 0.57 | 0.31 | 0.34 | -0.052 | -0.41 | -0.57 | 0.56 | -0.54 | 0.75 | -0.034 |
|  |  |  |  | GAIT SPEED | 0.45 | -0.77 (-1.6) | -0.71 | 0.14 | -0.18 | 0.32 | 0.51 | 0.54 | -0.13 | 0.35 | -0.63 | 0.1 |
|  |  |  |  | FGA | 0.56 | -0.91 (-0.076) | -0.64 | -0.36 | -0.36 | 0.077 | 0.5 | 0.48 | -0.49 | 0.45 | -0.68 | 0.22 |
|  | Physiological (VHIT) | IPSILESIONAL | MEAN | Horizontal | 0.79 | -0.34 | 0.24 | -0.2 | -0.53 | 0.088 | 0.17 | -0.16 | -0.4 | 0.23 | -0.54 | -0.39 |
|  |  |  |  | Posterior | 0.53 | -0.37 | -0.052 | 0.033 | -0.49 | 0.069 | -0.059 | 0.59 | -0.55 | 0.56 | -0.66 | -0.71 |
|  |  |  |  | Anterior | -0.93 (-420) | 0.7 | 0.47 | -0.36 | 0.68 | -0.71 | -0.69 | 0.16 | -0.34 | 0.26 | 0.32 | -0.29 |
|  |  |  | STANDARD  DEVIATION | Horizontal | 0.79 | -0.36 | -0.026 | 0.32 | -0.31 | 0.48 | 0.074 | 0.088 | -0.038 | 0.065 | -0.44 | -0.53 |
|  |  |  |  | Posterior | 0.7 | -0.5 | -0.24 | 0.27 | -0.58 | 0.37 | 0.011 | 0.55 | -0.29 | 0.3 | -0.54 | -0.6 |
|  |  |  |  | Anterior | -0.12 | 0.93 | 1 (430) | -0.37 | 0.87 | -0.7 | -0.28 | -0.35 | -0.65 | 0.81 | -0.98 (-11) | -0.58 |
|  |  | CONTRALESIONAL | MEAN | Horizontal | 0.36 | -0.71 | -0.92 (-34) | 0.17 | -0.35 | 0.71 | 0.55 | 0.17 | 0.42 | -0.45 | 0.18 | 0.7 |
|  |  |  |  | Posterior | 0.68 | -0.25 | -0.29 | 0.88 (0.11) | -0.19 | 0.93 (0.16) | 0.087 | -0.28 | 0.76 | -0.71 | 0.24 | 0.0068 |
|  |  |  |  | Anterior | -0.66 | -0.081 | -0.31 | -0.69 | -0.044 | -0.48 | 0.015 | 0.52 | -0.46 | 0.32 | 0.11 | 0.37 |
|  |  |  | STANDARD  DEVIATION | Horizontal | -0.035 | 0.054 | -0.19 | 0.19 | 0.052 | 0.41 | 0.22 | -0.63 | 0.75 | -0.78 | 0.63 | 0.75 |
|  |  |  |  | Posterior | -0.38 | 0.82 | 0.87 | 0.15 | 0.72 | -0.44 | -0.79 | -0.18 | -0.16 | 0.2 | 0.074 | -0.83 |
|  |  |  |  | Anterior | 0.16 | 0.1 | 0.45 | -0.36 | 0.046 | -0.39 | 0.55 | -0.72 | -0.057 | 0.21 | -0.41 | 0.32 |
|  | Subjective |  |  | DHI | -0.048 | 0.6 | 0.47 | 0.65 | 0.74 | 0.058 | -0.74 | -0.082 | 0.37 | -0.2 | 0.25 | -0.58 |
|  |  |  |  | ABC | 0.12 | -0.66 | -0.49 | -0.58 | -0.6 | -0.12 | 0.73 | 0.23 | -0.46 | 0.4 | -0.48 | 0.47 |
|  |  |  |  | HEADACHE | 0.45 | -0.011 | 0.17 | 0.54 | 0.58 | 0.044 | -0.22 | 0.19 | 0.032 | 0.33 | -0.57 | -0.55 |
|  |  |  |  | BECK ANXIETY | 0.48 | 0.048 | 0.23 | 0.5 | 0.59 | 0.072 | -0.53 | 0.27 | -0.044 | 0.24 | -0.39 | -0.73 |
|  |  |  |  |  | Peak  Vel. | Cycle  Dur. | Mov.  range | Peak  Vel. | Cycle  Dur. | Mov.  range | Peak  Vel. | Cycle  Dur. | Mov.  range | Peak  Vel. | Cycle  Dur. | Mov.  range |
|  |  |  |  |  | Mean | | | CV | | | Mean | | | CV | | |
|  |  |  |  |  |  | | | | | | Asymmetry | | | | | |
|  |  |  |  |  | Kinematic Measures | | | | | | | | | | | |

| Supplementary Table 8  Correlation coefficients (slope) for Task 2 (Postop. Clinical vs. Postop. Kinematics) | | | | | | | | | | | | | | | | |
| --- | --- | --- | --- | --- | --- | --- | --- | --- | --- | --- | --- | --- | --- | --- | --- | --- |
| Clinical Measures | Functional |  | DVA | IPSI | -0.3 | 0.39 | 0.39 | -0.25 | 0.32 | -0.12 | 0.75 | -0.79 (-0.2) | 0.17 | 0.49 | 0.15 | -0.49 |
|  |  |  |  | CONTRA | -0.51 | 0.79 (2.5) | 0.48 | -0.56 | 0.28 | -0.22 | 0.057 | -0.46 | -0.6 | 0.5 | -0.069 | 0.042 |
|  |  |  | TUG | IPSI | -0.22 | 0.61 | 0.61 | 0.033 | 0.15 | -0.25 | 0.61 | -0.47 | 0.0084 | 0.85 (0.053) | -0.33 | -0.44 |
|  |  |  |  | CONTRA | -0.24 | 0.59 | 0.59 | 0.02 | 0.18 | -0.21 | 0.63 | -0.49 | 0.057 | 0.83 (0.057) | -0.29 | -0.44 |
|  |  |  |  | GAIT SPEED | 0.057 | -0.83 (-1.5) | -0.9 (-25) | 0.28 | 0.16 | 0.61 | -0.44 | 0.5 | 0.16 | -0.93 (-1.3) | 0.36 | 0.089 |
|  |  |  |  | FGA | 0.33 | -0.66 | -0.57 | -0.043 | -0.2 | 0.2 | -0.5 | 0.39 | 0.13 | -0.83 (-0.048) | 0.33 | 0.42 |
|  | Physiological (VHIT) | IPSILESIONAL | MEAN | Horizontal | 0.18 | 0.15 | 0.051 | 0.16 | -0.42 | -0.091 | -0.8 | 0.85 (0.18) | -0.35 | 0.059 | -0.53 | 0.69 |
|  |  |  |  | Posterior | -0.46 | -0.033 | -0.48 | 0.2 | -0.6 | 0.36 | -0.89 (-0.65) | 0.84 | -0.68 | -0.41 | -0.084 | 0.41 |
|  |  |  |  | Anterior | -0.67 | 0.77 | 0.74 | -0.64 | 0.37 | -0.99 (-0.52) | 0.19 | -0.66 | -0.54 | 0.44 | 0.096 | -0.024 |
|  |  |  | STANDARD  DEVIATION | Horizontal | -0.062 | -0.16 | -0.34 | 0.6 | -0.17 | 0.28 | -0.68 | 0.98 (1.3) | -0.29 | -0.15 | -0.43 | 0.22 |
|  |  |  |  | Posterior | -0.31 | -0.26 | -0.64 | 0.48 | -0.71 | 0.56 | -0.76 | 0.93 (0.89) | -0.47 | -0.46 | -0.064 | 0.2 |
|  |  |  |  | Anterior | -0.46 | 0.91 | 0.82 | -0.53 | 0.7 | -0.33 | -0.81 | 0.17 | -0.64 | 0.95 (8.5) | -0.74 | 0.96 (6) |
|  |  | CONTRALESIONAL | MEAN | Horizontal | 0.57 | -0.87 (-1.7) | -0.55 | 0.52 | -0.13 | 0.29 | 0.39 | 0.052 | 0.79 | -0.55 | 0.59 | -0.46 |
|  |  |  |  | Posterior | 0.3 | -0.48 | -0.42 | 0.96 (0.19) | -0.21 | 0.71 | 0.16 | 0.55 | 0.43 | 0.034 | -0.32 | -0.5 |
|  |  |  |  | Anterior | -0.042 | -0.017 | 0.065 | -0.63 | -0.18 | -0.57 | 0.24 | -0.67 | -0.024 | -0.35 | 0.82 | -0.012 |
|  |  |  | STANDARD  DEVIATION | Horizontal | 0.6 | -0.25 | 0.15 | 0.26 | -0.00019 | -0.033 | 0.85 (3.4) | -0.46 | 0.9 (3.1) | 0.28 | -0.001 | -0.51 |
|  |  |  |  | Posterior | -0.84 | 0.94 (11) | 0.72 | -0.2 | 0.49 | -0.57 | -0.23 | 0.019 | -0.78 | 0.74 | -0.67 | 0.15 |
|  |  |  |  | Anterior | 0.51 | 0.11 | 0.25 | -0.41 | 0.64 | 0.18 | -0.13 | -0.07 | 0.29 | 0.22 | -0.47 | 0.63 |
|  | Subjective |  |  | DHI | 0.18 | 0.2 | 0.3 | 0.3 | 0.31 | -0.1 | 0.36 | -0.17 | -0.15 | 0.5 | -0.78 (-0.01) | -0.52 |
|  |  |  |  | ABC | -0.11 | -0.34 | -0.44 | -0.32 | -0.17 | 0.22 | -0.47 | 0.22 | 0.1 | -0.67 | 0.69 | 0.56 |
|  |  |  |  | HEADACHE | 0.32 | -0.34 | -0.31 | 0.27 | 0.44 | 0.35 | -0.26 | 0.31 | -0.11 | -0.18 | -0.72 | -0.025 |
|  |  |  |  | BECK ANXIETY | 0.4 | -0.16 | -0.074 | 0.32 | 0.21 | 0.051 | -0.2 | 0.29 | -0.29 | -0.00085 | -0.82 (-0.067) | -0.13 |
|  |  |  |  |  | Peak  Vel. | Cycle  Dur. | Mov.  range | Peak  Vel. | Cycle  Dur. | Mov.  range | Peak  Vel. | Cycle  Dur. | Mov.  range | Peak  Vel. | Cycle  Dur. | Mov.  range |
|  |  |  |  |  | Mean | | | CV | | | Mean | | | CV | | |
|  |  |  |  |  |  | | | | | | Asymmetry | | | | | |
|  |  |  |  |  | Kinematic Measures | | | | | | | | | | | |

| Supplementary Table 9  Correlation coefficients (slope) for Task 3 (Postop. Clinical vs. Postop. Kinematics) | | | | | | | | | | | | | | | | |
| --- | --- | --- | --- | --- | --- | --- | --- | --- | --- | --- | --- | --- | --- | --- | --- | --- |
| Clinical Measures | Functional |  | DVA | IPSI | -0.77 (-260) | 0.61 | -0.016 | 0.094 | 0.3 | 0.058 | -0.41 | -0.45 | 0.29 | 0.76 (1.3) | 0.66 | 0.74 |
|  |  |  |  | CONTRA | -0.9 (-320) | 0.78 (2.4) | 0.19 | 0.2 | 0.31 | -0.0045 | -0.45 | -0.097 | -0.16 | 0.67 | 0.27 | 0.79 (1.1) |
|  |  |  | TUG | IPSI | -0.55 | 0.86 (0.066) | 0.42 | 0.19 | 0.074 | -0.14 | -0.68 | -0.33 | -0.036 | 0.73 | 0.9 (0.11) | 0.54 |
|  |  |  |  | CONTRA | -0.56 | 0.85 (0.071) | 0.39 | 0.21 | 0.1 | -0.11 | -0.65 | -0.37 | 0.0074 | 0.73 | 0.89 (0.12) | 0.55 |
|  |  |  |  | GAIT SPEED | 0.45 | -0.93 (-1.6) | -0.69 | 0.14 | 0.22 | 0.51 | 0.58 | 0.1 | 0.44 | -0.46 | -0.75 | -0.72 |
|  |  |  |  | FGA | 0.62 | -0.89 (-0.064) | -0.4 | -0.28 | -0.13 | 0.084 | 0.7 | 0.33 | 0.028 | -0.79 (-0.033) | -0.86 (-0.1) | -0.54 |
|  | Physiological (VHIT) | IPSILESIONAL | MEAN | Horizontal | 0.64 | -0.068 | 0.52 | 0.014 | -0.44 | -0.32 | 0.27 | 0.73 | -0.65 | -0.66 | -0.34 | -0.5 |
|  |  |  |  | Posterior | 0.29 | -0.28 | -0.06 | 0.27 | -0.42 | 0.41 | 0.29 | 0.75 | -0.32 | -0.32 | -0.56 | -0.43 |
|  |  |  |  | Anterior | -0.98 (-460) | 0.69 | 0.19 | -0.26 | 0.81 | -0.91 (-0.23) | -0.48 | 0.06 | -0.31 | 0.68 | 0.24 | 0.97 (1.9) |
|  |  |  | STANDARD  DEVIATION | Horizontal | 0.59 | -0.25 | 0.16 | 0.3 | -0.19 | 0.058 | 0.14 | 0.41 | -0.18 | -0.36 | -0.23 | -0.75 |
|  |  |  |  | Posterior | 0.49 | -0.42 | -0.16 | 0.29 | -0.67 | 0.63 | 0.32 | 0.54 | -0.078 | -0.34 | -0.45 | -0.62 |
|  |  |  |  | Anterior | -0.35 | 0.95 (13) | 0.91 | 0.61 | 0.67 | -0.62 | -0.86 | 0.92 | -1 (-3.9) | -0.13 | -0.72 | 0.18 |
|  |  | CONTRALESIONAL | MEAN | Horizontal | 0.53 | -0.76 | -0.5 | -0.27 | -0.13 | 0.15 | 0.55 | -0.5 | 0.61 | -0.29 | -0.059 | -0.45 |
|  |  |  |  | Posterior | 0.65 | -0.23 | 0.049 | 0.48 | -0.76 | 0.68 | -0.11 | -0.46 | 0.44 | -0.041 | 0.39 | -0.74 |
|  |  |  |  | Anterior | -0.5 | -0.13 | -0.6 | -0.92 (-0.36) | 0.37 | -0.37 | 0.33 | 0.014 | 0.24 | 0.11 | -0.27 | 0.68 |
|  |  |  | STANDARD  DEVIATION | Horizontal | 0.18 | 0.032 | 0.058 | -0.13 | -0.1 | -0.085 | -0.15 | -0.82 (-0.81) | 0.3 | 0.076 | 0.59 | -0.013 |
|  |  |  |  | Posterior | -0.63 | 0.87 | 0.78 | 0.6 | 0.56 | -0.66 | -0.78 | 0.28 | -0.71 | 0.58 | 0.35 | 0.43 |
|  |  |  |  | Anterior | 0.25 | 0.082 | 0.5 | 0.34 | 0.38 | -0.12 | 0.071 | 0.21 | -0.53 | -0.51 | -0.19 | -0.2 |
|  | Subjective |  |  | DHI | -0.19 | 0.45 | 0.24 | 0.31 | 0.23 | 0.1 | -0.95 (-0.003) | 0.27 | -0.1 | 0.62 | 0.6 | -0.11 |
|  |  |  |  | ABC | 0.23 | -0.6 | -0.37 | -0.22 | -0.083 | 0.056 | 0.92 (0.003) | -0.11 | 0.12 | -0.66 | -0.79 (-0.024) | -0.068 |
|  |  |  |  | HEADACHE | 0.29 | -0.26 | -0.1 | 0.47 | 0.39 | 0.44 | -0.45 | 0.54 | -0.054 | -0.016 | -0.21 | -0.77 (-0.02) |
|  |  |  |  | BECK ANXIETY | 0.28 | -0.093 | 0.086 | 0.26 | 0.17 | 0.18 | -0.62 | 0.66 | -0.24 | 0.082 | -0.026 | -0.63 |
|  |  |  |  |  | Peak  Vel. | Cycle  Dur. | Mov.  range | Peak  Vel. | Cycle  Dur. | Mov.  range | Peak  Vel. | Cycle  Dur. | Mov.  range | Peak  Vel. | Cycle  Dur. | Mov.  range |
|  |  |  |  |  | Mean | | | CV | | | Mean | | | CV | | |
|  |  |  |  |  |  | | | | | | Asymmetry | | | | | |
|  |  |  |  |  | Kinematic Measures | | | | | | | | | | | |

| Supplementary Table 10  Correlation coefficients (slope) for Task 4 (Postop. Clinical vs. Postop. Kinematics) | | | | | | | | | | | | | | | | |
| --- | --- | --- | --- | --- | --- | --- | --- | --- | --- | --- | --- | --- | --- | --- | --- | --- |
| Clinical Measures | Functional |  | DVA | IPSI | -0.17 | 0.37 | 0.39 | 0.39 | 0.6 | 0.5 | 0.62 | -0.74 | -0.46 | 0.47 | 0.49 | 0.33 |
|  |  |  |  | CONTRA | -0.57 | 0.73 | 0.35 | 0.26 | 0.49 | 0.33 | 0.093 | -0.4 | -0.47 | -0.027 | 0.17 | -0.44 |
|  |  |  | TUG | IPSI | -0.23 | 0.62 | 0.57 | 0.75 | 0.51 | 0.38 | 0.78 (0.013) | -0.9 (-0.008) | -0.73 | 0.03 | 0.82 (0.024) | 0.21 |
|  |  |  |  | CONTRA | -0.22 | 0.6 | 0.56 | 0.76 (0.004) | 0.55 | 0.41 | 0.77 (0.014) | -0.91 (-0.009) | -0.73 | 0.062 | 0.83 (0.027) | 0.26 |
|  |  |  |  | GAIT SPEED | 0.033 | -0.82 (-1.5) | -0.85 (-25) | -0.54 | -0.32 | 0.032 | -0.69 | 0.87 (0.18) | 0.44 | -0.053 | -0.68 | 0.039 |
|  |  |  |  | FGA | 0.38 | -0.66 | -0.5 | -0.75 | -0.5 | -0.44 | -0.68 | 0.82 (0.007) | 0.79 (0.006) | 0.052 | -0.78 (-0.022) | -0.076 |
|  | Physiological (VHIT) | IPSILESIONAL | MEAN | Horizontal | 0.014 | 0.2 | 0.066 | 0.039 | -0.42 | -0.56 | -0.45 | 0.33 | 0.1 | -0.7 | -0.061 | -0.46 |
|  |  |  |  | Posterior | -0.6 | -0.031 | -0.67 | -0.34 | -0.69 | -0.44 | -0.78 | 0.8 | 0.16 | -0.55 | -0.54 | -0.78 |
|  |  |  |  | Anterior | -0.46 | 0.73 | 0.62 | -0.034 | 0.37 | 0.31 | 0.17 | -0.33 | -0.18 | 0.26 | 0.001 | -0.44 |
|  |  |  | STANDARD  DEVIATION | Horizontal | -0.27 | -0.11 | -0.35 | 0.099 | -0.43 | -0.16 | -0.41 | 0.49 | -0.11 | -0.71 | -0.052 | -0.34 |
|  |  |  |  | Posterior | -0.49 | -0.24 | -0.8 | -0.29 | -0.8 | -0.33 | -0.65 | 0.79 | 0.14 | -0.5 | -0.43 | -0.57 |
|  |  |  |  | Anterior | -0.45 | 0.93 | 0.65 | 0.53 | 0.59 | -0.73 | -0.79 | -0.26 | -0.65 | -0.75 | 0.11 | -0.58 |
|  |  | CONTRALESIONAL | MEAN | Horizontal | 0.55 | -0.84 (-1.7) | -0.38 | -0.36 | -0.35 | -0.027 | 0.14 | 0.25 | 0.46 | 0.56 | -0.14 | 0.65 |
|  |  |  |  | Posterior | 0.74 | -0.41 | -0.36 | 0.42 | -0.28 | 0.36 | 0.33 | -0.041 | -0.35 | -0.26 | 0.48 | 0.45 |
|  |  |  |  | Anterior | 0.19 | -0.093 | 0.067 | -0.7 | -0.064 | -0.2 | -0.11 | 0.14 | 0.63 | 0.8 | -0.55 | -0.1 |
|  |  |  | STANDARD  DEVIATION | Horizontal | 0.63 | -0.22 | 0.3 | 0.39 | 0.27 | 0.12 | 0.78 | -0.67 | -0.12 | 0.43 | 0.63 | 0.93 (12) |
|  |  |  |  | Posterior | -0.83 | 0.95 (10) | 0.53 | 0.52 | 0.38 | 0.39 | 0.077 | -0.34 | -0.74 | -0.58 | 0.36 | -0.62 |
|  |  |  |  | Anterior | 0.51 | 0.12 | 0.41 | 0.39 | 0.67 | -0.39 | -0.082 | -0.33 | -0.072 | -0.39 | 0.2 | 0.32 |
|  | Subjective |  |  | DHI | -0.19 | 0.18 | 0.073 | 0.66 | 0.3 | 0.39 | 0.56 | -0.45 | -0.6 | -0.49 | 0.47 | 0.093 |
|  |  |  |  | ABC | 0.18 | -0.33 | -0.27 | -0.71 | -0.27 | -0.34 | -0.72 | 0.62 | 0.64 | 0.34 | -0.65 | -0.13 |
|  |  |  |  | HEADACHE | -0.062 | -0.36 | -0.5 | 0.3 | 0.16 | 0.23 | -0.18 | 0.24 | -0.21 | -0.82 (-0.029) | -0.12 | 0.027 |
|  |  |  |  | BECK ANXIETY | -0.065 | -0.19 | -0.32 | 0.27 | -0.055 | 0.089 | -0.0029 | 0.17 | -0.18 | -0.79 (-0.064) | -0.056 | -0.15 |
|  |  |  |  |  | Peak  Vel. | Cycle  Dur. | Mov.  range | Peak  Vel. | Cycle  Dur. | Mov.  range | Peak  Vel. | Cycle  Dur. | Mov.  range | Peak  Vel. | Cycle  Dur. | Mov.  range |
|  |  |  |  |  | Mean | | | CV | | | Mean | | | CV | | |
|  |  |  |  |  |  | | | | | | Asymmetry | | | | | |
|  |  |  |  |  | Kinematic Measures | | | | | | | | | | | |

| Supplementary Table 11  Correlation coefficients (slope) for Task 5 (Postop. Clinical vs. Postop. Kinematics) | | | | | | | | | | | | | | | | |
| --- | --- | --- | --- | --- | --- | --- | --- | --- | --- | --- | --- | --- | --- | --- | --- | --- |
| Clinical Measures | Functional |  | DVA | IPSI | -0.86 (-250) | 0.64 | 0.028 | -0.52 | 0.46 | -0.2 | 0.032 | -0.54 | 0.35 | 0.11 | -0.64 | 0.6 |
|  |  |  |  | CONTRA | -0.83 (-260) | 0.65 | 0.23 | -0.78 (-0.38) | 0.59 | -0.21 | 0.058 | 0.071 | 0.048 | 0.66 | -0.56 | -0.17 |
|  |  |  | TUG | IPSI | -0.6 | 0.87 (0.1) | 0.34 | -0.3 | 0.4 | -0.28 | -0.17 | -0.44 | 0.28 | 0.5 | -0.57 | 0.55 |
|  |  |  |  | CONTRA | -0.62 | 0.87 (0.11) | 0.32 | -0.29 | 0.43 | -0.27 | -0.14 | -0.49 | 0.32 | 0.46 | -0.55 | 0.58 |
|  |  |  |  | GAIT SPEED | 0.47 | -0.96 (-2.6) | -0.64 | 0.55 | -0.39 | 0.66 | -0.0028 | 0.13 | 0.13 | -0.64 | 0.57 | -0.38 |
|  |  |  |  | FGA | 0.64 | -0.86 (-0.095) | -0.3 | 0.31 | -0.41 | 0.2 | 0.17 | 0.39 | -0.29 | -0.6 | 0.58 | -0.41 |
|  | Physiological (VHIT) | IPSILESIONAL | MEAN | Horizontal | 0.77 | -0.093 | 0.49 | 0.42 | -0.16 | -0.12 | 0.18 | 0.63 | -0.41 | 0.4 | 0.65 | -0.56 |
|  |  |  |  | Posterior | 0.51 | -0.48 | -0.18 | 0.26 | -0.41 | 0.65 | -0.027 | 0.76 | -0.64 | 0.31 | 0.31 | -0.89 (-2.7) |
|  |  |  |  | Anterior | -0.94 (-380) | 0.62 | 0.22 | -0.95 (-0.69) | 0.43 | -0.69 | 0.17 | 0.21 | -0.36 | 0.5 | -0.77 | -0.1 |
|  |  |  | STANDARD  DEVIATION | Horizontal | 0.71 | -0.33 | 0.012 | 0.72 | -0.38 | 0.37 | -0.28 | 0.34 | -0.089 | 0.24 | 0.53 | -0.5 |
|  |  |  |  | Posterior | 0.66 | -0.58 | -0.35 | 0.52 | -0.61 | 0.85 | -0.19 | 0.53 | -0.46 | 0.14 | 0.35 | -0.71 |
|  |  |  |  | Anterior | -0.17 | 0.94 | 0.89 | -0.38 | 0.78 | -0.44 | 0.084 | 0.82 | -0.17 | 0.88 | 0.31 | -0.71 |
|  |  | CONTRALESIONAL | MEAN | Horizontal | 0.39 | -0.61 | -0.53 | 0.59 | -0.6 | 0.26 | 0.051 | -0.56 | 0.11 | -0.9 (-1.1) | 0.2 | 0.49 |
|  |  |  |  | Posterior | 0.6 | -0.17 | -0.25 | 0.91 (0.22) | -0.5 | 0.72 | -0.75 | -0.54 | 0.6 | -0.18 | 0.25 | 0.35 |
|  |  |  |  | Anterior | -0.55 | -0.13 | -0.36 | -0.71 | -0.013 | -0.46 | 0.73 | 0.12 | -0.56 | -0.35 | -0.44 | 0.0098 |
|  |  |  | STANDARD  DEVIATION | Horizontal | -0.024 | 0.24 | 0.064 | 0.24 | 0.04 | -0.2 | -0.11 | -0.88 (-1.6) | 0.35 | -0.45 | -0.069 | 0.97 (16) |
|  |  |  |  | Posterior | -0.48 | 0.74 | 0.6 | -0.44 | 0.48 | -0.29 | -0.42 | 0.36 | -0.074 | 0.99 (7.3) | -0.39 | -0.36 |
|  |  |  |  | Anterior | 0.2 | 0.18 | 0.78 | 0.042 | 0.75 | -0.47 | 0.27 | 0.068 | 0.42 | 0.024 | 0.65 | 0.087 |
|  | Subjective |  |  | DHI | -0.21 | 0.37 | -0.022 | -0.049 | 0.15 | 0.16 | -0.82 (-0.002) | 0.018 | 0.35 | 0.44 | -0.48 | 0.2 |
|  |  |  |  | ABC | 0.26 | -0.56 | -0.11 | 0.074 | -0.12 | 0.0085 | 0.67 | 0.13 | -0.27 | -0.49 | 0.56 | -0.35 |
|  |  |  |  | HEADACHE | 0.31 | -0.36 | -0.24 | 0.35 | 0.1 | 0.56 | -0.77 (-0.005) | 0.36 | 0.4 | 0.072 | 0.26 | -0.3 |
|  |  |  |  | BECK ANXIETY | 0.33 | -0.2 | -0.16 | 0.22 | -0.092 | 0.38 | -0.87 (-0.012) | 0.48 | 0.16 | 0.26 | -0.017 | -0.3 |
|  |  |  |  |  | Peak  Vel. | Cycle  Dur. | Mov.  range | Peak  Vel. | Cycle  Dur. | Mov.  range | Peak  Vel. | Cycle  Dur. | Mov.  range | Peak  Vel. | Cycle  Dur. | Mov.  range |
|  |  |  |  |  | Mean | | | CV | | | Mean | | | CV | | |
|  |  |  |  |  |  | | | | | | Asymmetry | | | | | |
|  |  |  |  |  | Kinematic Measures | | | | | | | | | | | |

| Supplementary Table 12  Correlation coefficients (slope) for Task 6 (Postop. Clinical vs. Postop. Kinematics) | | | | | | | | | | | | | | | | |
| --- | --- | --- | --- | --- | --- | --- | --- | --- | --- | --- | --- | --- | --- | --- | --- | --- |
| Clinical Measures | Functional |  | DVA | IPSI | -0.4 | 0.5 | 0.34 | -0.15 | -0.063 | -0.12 | 0.31 | -0.46 | -0.22 | -0.019 | 0.27 | 0.39 |
|  |  |  |  | CONTRA | -0.66 | 0.7 | 0.41 | -0.067 | 0.32 | -0.13 | -0.37 | 0.19 | -0.52 | -0.46 | 0.057 | -0.032 |
|  |  |  | TUG | IPSI | -0.36 | 0.75 | 0.58 | 0.061 | 0.0092 | -0.27 | 0.2 | -0.4 | -0.35 | -0.39 | -0.06 | -0.12 |
|  |  |  |  | CONTRA | -0.36 | 0.73 | 0.57 | 0.078 | 0.022 | -0.25 | 0.24 | -0.43 | -0.32 | -0.38 | -0.04 | -0.086 |
|  |  |  |  | GAIT SPEED | 0.15 | -0.91 (-2.2) | -0.86 (-27) | 0.19 | -0.15 | 0.63 | -0.25 | 0.43 | 0.18 | 0.44 | 0.42 | 0.22 |
|  |  |  |  | FGA | 0.49 | -0.76 (-0.074) | -0.54 | -0.13 | -0.028 | 0.2 | -0.04 | 0.24 | 0.49 | 0.46 | 0.0047 | 0.16 |
|  | Physiological (VHIT) | IPSILESIONAL | MEAN | Horizontal | 0.28 | 0.072 | 0.17 | 0.46 | 0.33 | -0.16 | -0.32 | 0.36 | 0.059 | -0.51 | -0.64 | -0.77 |
|  |  |  |  | Posterior | -0.32 | -0.25 | -0.49 | 0.39 | 0.011 | 0.66 | -0.88 (-1) | 0.96 (0.58) | -0.45 | -0.18 | 0.029 | -0.33 |
|  |  |  |  | Anterior | -0.7 | 0.71 | 0.58 | -0.61 | 0.12 | -0.84 | -0.22 | 0.086 | -0.44 | -0.15 | 0.1 | 0.21 |
|  |  |  | STANDARD  DEVIATION | Horizontal | 0.0036 | -0.22 | -0.25 | 0.69 | -0.011 | 0.24 | -0.48 | 0.54 | -0.26 | -0.31 | -0.15 | -0.59 |
|  |  |  |  | Posterior | -0.19 | -0.41 | -0.65 | 0.49 | -0.19 | 0.83 | -0.73 | 0.83 | -0.42 | -0.043 | 0.16 | -0.27 |
|  |  |  |  | Anterior | -0.39 | 0.95 (19) | 0.86 | 0.38 | 0.89 | -0.37 | -0.59 | 0.48 | -0.11 | -0.94 | -0.81 | -0.8 |
|  |  | CONTRALESIONAL | MEAN | Horizontal | 0.54 | -0.74 | -0.51 | -0.14 | -0.64 | 0.19 | 0.56 | -0.4 | 0.41 | 0.83 (0.53) | 0.37 | 0.61 |
|  |  |  |  | Posterior | 0.23 | -0.31 | -0.34 | 0.67 | -0.43 | 0.63 | 0.19 | -0.15 | -0.077 | 0.053 | 0.14 | -0.21 |
|  |  |  |  | Anterior | -0.031 | -0.1 | -0.087 | -0.98 (-0.35) | -0.18 | -0.46 | 0.18 | -0.13 | 0.12 | 0.59 | 0.44 | 0.79 |
|  |  |  | STANDARD  DEVIATION | Horizontal | 0.48 | -0.0046 | 0.2 | -0.099 | -0.23 | -0.17 | 0.88 (6.3) | -0.91 (-3.3) | 0.5 | 0.27 | -0.044 | 0.24 |
|  |  |  |  | Posterior | -0.84 | 0.88 (12) | 0.68 | 0.27 | 0.38 | -0.43 | -0.64 | 0.44 | -0.67 | -0.79 | -0.34 | -0.59 |
|  |  |  |  | Anterior | 0.56 | 0.14 | 0.46 | 0.35 | 0.85 | -0.17 | 0.29 | -0.34 | 0.74 | -0.53 | -0.91 (-1.2) | -0.56 |
|  | Subjective |  |  | DHI | -0.11 | 0.26 | 0.1 | 0.019 | -0.0063 | 0.06 | -0.15 | 0.061 | -0.26 | -0.3 | 0.037 | -0.47 |
|  |  |  |  | ABC | 0.14 | -0.43 | -0.29 | -0.011 | 0.099 | 0.11 | 0.012 | 0.11 | 0.32 | 0.31 | 0.028 | 0.39 |
|  |  |  |  | HEADACHE | 0.16 | -0.39 | -0.43 | 0.25 | 0.31 | 0.5 | -0.42 | 0.46 | 0.13 | -0.26 | 0.025 | -0.65 |
|  |  |  |  | BECK ANXIETY | 0.17 | -0.22 | -0.27 | 0.06 | 0.12 | 0.26 | -0.43 | 0.46 | -0.029 | -0.22 | -0.06 | -0.68 |
|  |  |  |  |  | Peak  Vel. | Cycle  Dur. | Mov.  range | Peak  Vel. | Cycle  Dur. | Mov.  range | Peak  Vel. | Cycle  Dur. | Mov.  range | Peak  Vel. | Cycle  Dur. | Mov.  range |
|  |  |  |  |  | Mean | | | CV | | | Mean | | | CV | | |
|  |  |  |  |  |  | | | | | | Asymmetry | | | | | |
|  |  |  |  |  | Kinematic Measures | | | | | | | | | | | |

| Supplementary Table 13  Correlation coefficients (slope) for Task 1 (Preop. Clinical vs. Postop. Kinematics) | | | | | | | | | | | | | | | | |
| --- | --- | --- | --- | --- | --- | --- | --- | --- | --- | --- | --- | --- | --- | --- | --- | --- |
| Clinical Measures | Functional |  | DVA | IPSI | -0.43 | 0.66 | 0.44 | 0.13 | 0.37 | -0.09 | -0.62 | -0.32 | 0.21 | -0.29 | 0.78 (1.6) | -0.15 |
|  |  |  |  | CONTRA | -0.54 | 0.58 | 0.2 | 0.24 | 0.35 | 0.081 | -0.43 | -0.35 | 0.35 | -0.31 | 0.89 (3.3) | 0.056 |
|  |  |  | TUG | IPSI | -0.42 | 0.65 | 0.49 | 0.11 | -0.037 | 0.1 | -0.29 | -0.58 | 0.36 | -0.44 | 0.86 (0.12) | -0.015 |
|  |  |  |  | CONTRA | -0.49 | 0.72 (0.14) | 0.55 | 0.14 | 0.034 | 0.035 | -0.27 | -0.62 | 0.34 | -0.36 | 0.84 (0.12) | -0.041 |
|  |  |  |  | GAIT SPEED | 0.67 | -0.72 (-1.3) | -0.5 | -0.05 | -0.45 | 0.32 | 0.33 | 0.49 | -0.0078 | -0.074 | -0.67 | 0.025 |
|  |  |  |  | FGA | 0.33 | -0.54 | -0.42 | -0.31 | -0.73 (-0.018) | 0.21 | 0.68 | 0.091 | 0.22 | -0.33 | -0.31 | 0.6 |
|  | Physiological (VHIT) | IPSILESIONAL | MEAN | Horizontal | 0.82 (170) | -0.52 | -0.069 | -0.048 | -0.17 | 0.25 | 0.21 | -0.079 | -0.081 | 0.014 | -0.52 | -0.0071 |
|  |  |  |  | Posterior | -0.23 | 0.18 | 0.033 | 0.4 | 0.27 | -0.059 | 0.48 | -0.11 | 0.46 | 0.073 | -0.3 | 0.23 |
|  |  |  |  | Anterior | 0.4 | -0.47 | -0.12 | -0.76 (-0.15) | -0.15 | -0.41 | 0.038 | 0.16 | -0.51 | 0.11 | -0.38 | 0.36 |
|  |  |  | STANDARD  DEVIATION | Horizontal | -0.54 | 0.75 (4.6) | 0.41 | 0.41 | 0.24 | 0.18 | -0.28 | -0.55 | 0.6 | -0.45 | 0.89 (4.1) | 0.018 |
|  |  |  |  | Posterior | 0.53 | -0.49 | -0.37 | -0.028 | -0.44 | 0.68 | 0.25 | -0.34 | 0.13 | -0.33 | 0.29 | 0.35 |
|  |  |  |  | Anterior | -0.62 | 0.88 (8.7) | 0.62 | 0.32 | 0.51 | -0.16 | -0.47 | -0.47 | 0.35 | -0.19 | 0.71 (5.2) | -0.19 |
|  |  | CONTRALESIONAL | MEAN | Horizontal | 0.39 | -0.27 | -0.43 | 0.37 | 0.087 | 0.72 (0.21) | -0.13 | -0.096 | 0.55 | -0.55 | 0.42 | 0.26 |
|  |  |  |  | Posterior | 0.63 | -0.5 | -0.23 | 0.088 | 0.23 | 0.12 | 0.064 | 0.17 | -0.21 | 0.29 | -0.59 | -0.11 |
|  |  |  |  | Anterior | -0.27 | 0.34 | 0.29 | 0.35 | 0.69 | -0.26 | 0.19 | -0.37 | 0.12 | 0.39 | -0.15 | 0.067 |
|  |  |  | STANDARD  DEVIATION | Horizontal | 0.012 | 0.094 | -0.1 | 0.86 (2.7) | 0.62 | 0.37 | 0.043 | -0.033 | 0.34 | 0.24 | -0.14 | -0.31 |
|  |  |  |  | Posterior | -0.34 | 0.5 | 0.3 | 0.23 | -0.031 | 0.33 | 0.0068 | -0.78 (-0.49) | 0.53 | -0.47 | 0.9 (4.3) | 0.28 |
|  |  |  |  | Anterior | -0.13 | 0.016 | -0.29 | 0.78 (1.3) | 0.34 | 0.57 | 0.19 | -0.19 | 0.24 | 0.24 | 0.21 | -0.16 |
|  | Subjective |  |  | DHI | 0.11 | 0.13 | -0.035 | 0.6 | 0.42 | 0.48 | -0.53 | -0.011 | 0.15 | -0.044 | 0.37 | -0.52 |
|  |  |  |  | ABC | -0.47 | 0.15 | 0.018 | -0.41 | -0.27 | -0.4 | 0.38 | -0.053 | 0.12 | -0.15 | 0.07 | 0.59 |
|  |  |  |  | HEADACHE | -0.15 | 0.58 | 0.51 | 0.5 | 0.4 | 0.22 | -0.1 | -0.76 (-0.005) | 0.53 | -0.24 | 0.52 | -0.019 |
|  |  |  |  | BECK ANXIETY | 0.1 | 0.18 | 0.043 | 0.75 (0.007) | -0.03 | 0.69 | -0.025 | -0.19 | 0.59 | -0.31 | 0.25 | -0.36 |
|  |  |  |  |  | Peak  Vel. | Cycle  Dur. | Mov.  range | Peak  Vel. | Cycle  Dur. | Mov.  range | Peak  Vel. | Cycle  Dur. | Mov.  range | Peak  Vel. | Cycle  Dur. | Mov.  range |
|  |  |  |  |  | Mean | | | CV | | | Mean | | | CV | | |
|  |  |  |  |  |  | | | | | | Asymmetry | | | | | |
|  |  |  |  |  | Kinematic Measures | | | | | | | | | | | |

| Supplementary Table 14  Correlation coefficients (slope) for Task 2 (Preop. Clinical vs. Postop. Kinematics) | | | | | | | | | | | | | | | | |
| --- | --- | --- | --- | --- | --- | --- | --- | --- | --- | --- | --- | --- | --- | --- | --- | --- |
| Clinical Measures | Functional |  | DVA | IPSI | 0.033 | 0.58 | 0.79 (28) | -0.031 | -0.097 | -0.6 | 0.68 | -0.62 | -0.14 | 0.78 (1.4) | -0.35 | -0.38 |
|  |  |  |  | CONTRA | -0.55 | 0.41 | 0.61 | 0.088 | 0.0011 | -0.43 | 0.82 (0.62) | -0.7 | -0.027 | 0.62 | -0.13 | -0.57 |
|  |  |  | TUG | IPSI | -0.32 | 0.74 (0.12) | 0.74 (1.8) | 0.12 | -0.2 | -0.44 | 0.47 | -0.3 | -0.15 | 0.9 (0.11) | -0.16 | -0.31 |
|  |  |  |  | CONTRA | -0.36 | 0.78 (0.14) | 0.75 (1.9) | 0.067 | -0.12 | -0.4 | 0.46 | -0.35 | -0.19 | 0.91 (0.12) | -0.19 | -0.34 |
|  |  |  |  | GAIT SPEED | 0.1 | -0.64 | -0.77 (-18) | 0.29 | -0.1 | 0.49 | -0.61 | 0.81 (0.11) | 0.26 | -0.66 | 0.27 | 0.53 |
|  |  |  |  | FGA | -0.089 | -0.43 | -0.55 | 0.024 | -0.15 | 0.44 | -0.29 | 0.48 | 0.56 | -0.4 | 0.62 | 0.59 |
|  | Physiological (VHIT) | IPSILESIONAL | MEAN | Horizontal | 0.53 | -0.37 | -0.16 | 0.27 | -0.23 | 0.063 | -0.38 | 0.56 | 0.23 | -0.16 | -0.47 | 0.26 |
|  |  |  |  | Posterior | -0.29 | -0.34 | -0.59 | -0.022 | 0.8 (0.32) | 0.86 (0.39) | 0.0094 | -0.022 | 0.32 | -0.29 | 0.11 | 0.058 |
|  |  |  |  | Anterior | 0.77 (140) | -0.2 | 0.24 | -0.56 | -0.43 | -0.5 | -0.012 | -0.22 | 0.33 | -0.15 | -0.07 | 0.44 |
|  |  |  | STANDARD  DEVIATION | Horizontal | -0.34 | 0.53 | 0.53 | 0.23 | 0.13 | -0.17 | 0.68 | -0.44 | -0.014 | 0.78 (3.1) | -0.17 | -0.46 |
|  |  |  |  | Posterior | 0.36 | -0.21 | 0.12 | 0.55 | -0.59 | -0.26 | 0.15 | 0.27 | 0.28 | 0.058 | -0.071 | -0.25 |
|  |  |  |  | Anterior | -0.22 | 0.64 | 0.67 | -0.051 | 0.24 | -0.29 | 0.62 | -0.62 | -0.19 | 0.81 (5.2) | -0.39 | -0.43 |
|  |  | CONTRALESIONAL | MEAN | Horizontal | 0.52 | -0.47 | -0.035 | 0.67 | -0.19 | -0.088 | 0.62 | -0.047 | 0.54 | 0.04 | -0.2 | -0.33 |
|  |  |  |  | Posterior | 6.20E-01 | -0.56 | -0.3 | 0.17 | 0.032 | 0.11 | -0.22 | 0.25 | 0.14 | -0.44 | -0.45 | 0.016 |
|  |  |  |  | Anterior | 0.11 | -0.1 | -0.026 | -0.18 | 0.69 | 0.33 | 0.26 | -0.47 | 0.079 | -0.015 | -0.45 | -0.36 |
|  |  |  | STANDARD  DEVIATION | Horizontal | -0.064 | -0.41 | -0.5 | 0.55 | 0.67 | 0.63 | 0.062 | 0.1 | -0.063 | -0.3 | -0.37 | -0.54 |
|  |  |  |  | Posterior | -0.2 | 0.48 | 0.6 | 0.29 | -0.12 | -0.27 | 0.64 | -0.35 | 0.11 | 0.77 (3.2) | -0.15 | -0.5 |
|  |  |  |  | Anterior | -0.26 | -0.26 | -0.36 | 0.66 | 0.4 | 0.44 | 0.13 | 0.056 | -0.21 | -0.28 | -0.083 | -0.85 (-9) |
|  | Subjective |  |  | DHI | 0.13 | 0.0089 | 0.15 | 0.64 | -0.033 | -0.19 | 0.26 | 0.031 | -0.29 | 0.18 | -0.5 | -0.62 |
|  |  |  |  | ABC | -3.10E-01 | 0.095 | -0.049 | -0.57 | 0.16 | 0.16 | 0.15 | -0.35 | 0.33 | -0.011 | 0.63 | 0.36 |
|  |  |  |  | HEADACHE | 0.0071 | 0.33 | 0.47 | 0.32 | 0.22 | -0.058 | 0.5 | -0.26 | 0.072 | 0.7 | -0.65 | -0.48 |
|  |  |  |  | BECK ANXIETY | -0.47 | 0.031 | -0.27 | 0.84 (0.009) | 0.17 | 0.44 | -0.1 | 0.58 | -0.14 | 0.18 | -0.16 | -0.28 |
|  |  |  |  |  | Peak  Vel. | Cycle  Dur. | Mov.  range | Peak  Vel. | Cycle  Dur. | Mov.  range | Peak  Vel. | Cycle  Dur. | Mov.  range | Peak  Vel. | Cycle  Dur. | Mov.  range |
|  |  |  |  |  | Mean | | | CV | | | Mean | | | CV | | |
|  |  |  |  |  |  | | | | | | Asymmetry | | | | | |
|  |  |  |  |  | Kinematic Measures | | | | | | | | | | | |

| Supplementary Table 15  Correlation coefficients (slope) for Task 3 (Preop. Clinical vs. Postop. Kinematics) | | | | | | | | | | | | | | | | |
| --- | --- | --- | --- | --- | --- | --- | --- | --- | --- | --- | --- | --- | --- | --- | --- | --- |
| Clinical Measures | Functional |  | DVA | IPSI | -0.43 | 0.75 (1.8) | 0.46 | -0.17 | -0.15 | -0.4 | -0.55 | -0.13 | -0.25 | 0.62 | 0.85 (3.2) | 0.39 |
|  |  |  |  | CONTRA | -0.48 | 0.61 | 0.21 | -0.12 | -0.046 | -0.24 | -0.35 | -0.34 | 0.026 | 0.66 | 0.85 (5.5) | 0.29 |
|  |  |  | TUG | IPSI | -0.41 | 0.87 (0.14) | 0.6 | -0.011 | -0.27 | -0.43 | -0.25 | -0.43 | -0.2 | 0.5 | 0.9 (0.22) | 0.41 |
|  |  |  |  | CONTRA | -0.48 | 0.91 (0.15) | 0.58 | 0.058 | -0.19 | -0.37 | -0.25 | -0.46 | -0.2 | 0.54 | 0.88 (0.23) | 0.39 |
|  |  |  |  | GAIT SPEED | 0.63 | -0.74 (-1.1) | -0.31 | 0.042 | -0.059 | 0.24 | 0.26 | 0.33 | 0.24 | -0.54 | -0.64 | -0.27 |
|  |  |  |  | FGA | 0.43 | -0.48 | -0.19 | -0.025 | -0.12 | 0.1 | 0.47 | -0.013 | 0.34 | -0.53 | -0.41 | 0.12 |
|  | Physiological (VHIT) | IPSILESIONAL | MEAN | Horizontal | 0.78 (170) | -0.43 | 0.26 | -0.049 | -0.3 | -0.13 | 0.084 | 0.34 | -0.35 | -0.73 (-0.7) | -0.32 | -0.61 |
|  |  |  |  | Posterior | -0.2 | -0.19 | -0.52 | 0.71 | 0.77 (0.25) | 0.82 (0.42) | 0.017 | -0.2 | 0.63 | 0.18 | -0.19 | -0.2 |
|  |  |  |  | Anterior | 0.51 | -0.34 | 0.22 | -0.68 | -0.42 | -0.49 | 0.054 | 0.55 | -0.52 | -0.7 | -0.42 | 0.11 |
|  |  |  | STANDARD  DEVIATION | Horizontal | -0.52 | 0.78 (4) | 0.32 | 0.21 | 0.05 | -0.1 | -0.37 | -0.49 | 0.12 | 0.73 (2.1) | 0.96 (7.9) | 0.29 |
|  |  |  |  | Posterior | 0.59 | -0.22 | 0.31 | -0.37 | -0.66 | -0.47 | 0.38 | -0.17 | -0.19 | -0.53 | 0.2 | -0.45 |
|  |  |  |  | Anterior | -0.63 | 0.85 (7) | 0.35 | 0.2 | 0.17 | -0.092 | -0.56 | -0.29 | -0.088 | 0.77 (3.6) | 0.84 (11) | 0.32 |
|  |  | CONTRALESIONAL | MEAN | Horizontal | 0.45 | -0.26 | 0.0003 | -0.21 | -0.26 | -0.12 | -0.19 | 0.013 | 0.19 | -0.018 | 0.46 | -0.36 |
|  |  |  |  | Posterior | 0.59 | -0.61 | -0.12 | -0.041 | 0.0032 | 0.12 | 0.0067 | 0.41 | -0.18 | -0.53 | -0.45 | -0.72 (-1.1) |
|  |  |  |  | Anterior | -0.24 | 0.013 | -0.21 | 0.47 | 0.62 | 0.49 | -0.12 | -0.14 | 0.11 | 0.14 | -0.024 | -0.46 |
|  |  |  | STANDARD  DEVIATION | Horizontal | -0.081 | -0.26 | -0.49 | 0.65 | 0.62 | 0.74 (6) | -0.11 | -0.17 | 0.45 | 0.29 | 0.0037 | -0.79 (-13) |
|  |  |  |  | Posterior | -0.27 | 0.66 | 0.47 | 0.044 | -0.23 | -0.32 | -0.048 | -0.6 | -0.041 | 0.34 | 0.89 (7.5) | 0.098 |
|  |  |  |  | Anterior | -0.19 | -0.19 | -0.46 | 0.46 | 0.36 | 0.51 | 0.29 | -0.53 | 0.45 | 0.26 | 0.12 | -0.79 (-6.7) |
|  | Subjective |  |  | DHI | -0.008 | 0.12 | 0.072 | 0.047 | -0.074 | -0.037 | -0.32 | -0.05 | -0.055 | 0.36 | 0.47 | -0.45 |
|  |  |  |  | ABC | -0.32 | 0.11 | -0.18 | -0.0065 | 0.2 | 0.1 | 0.18 | -0.17 | 0.31 | 0.073 | -0.091 | 0.66 |
|  |  |  |  | HEADACHE | -0.16 | 0.56 | 0.43 | 0.35 | 0.073 | -0.028 | -0.34 | -0.35 | -0.12 | 0.33 | 0.75 (0.073) | -0.27 |
|  |  |  |  | BECK ANXIETY | -0.036 | 0.17 | 0.016 | 0.56 | 0.11 | 0.3 | -0.089 | -0.36 | 0.33 | 0.36 | 0.41 | -0.34 |
|  |  |  |  |  | Peak  Vel. | Cycle  Dur. | Mov.  range | Peak  Vel. | Cycle  Dur. | Mov.  range | Peak  Vel. | Cycle  Dur. | Mov.  range | Peak  Vel. | Cycle  Dur. | Mov.  range |
|  |  |  |  |  | Mean | | | CV | | | Mean | | | CV | | |
|  |  |  |  |  |  | | | | | | Asymmetry | | | | | |
|  |  |  |  |  | Kinematic Measures | | | | | | | | | | | |

| Supplementary Table 16  Correlation coefficients (slope) for Task 4 (Preop. Clinical vs. Postop. Kinematics) | | | | | | | | | | | | | | | | |
| --- | --- | --- | --- | --- | --- | --- | --- | --- | --- | --- | --- | --- | --- | --- | --- | --- |
| Clinical Measures | Functional |  | DVA | IPSI | -0.02 | 0.57 | 0.69 | 0.43 | 0.21 | 0.088 | 0.86 (0.43) | -0.73 (-0.2) | -0.35 | 0.15 | 0.53 | -0.077 |
|  |  |  |  | CONTRA | -0.26 | 0.4 | 0.57 | 0.4 | 0.27 | 0.23 | 0.89 (0.76) | -0.66 | -0.39 | 0.33 | 0.46 | -0.031 |
|  |  |  | TUG | IPSI | -0.22 | 0.77 (0.13) | 0.78 (2) | 0.56 | 0.21 | 0.082 | 0.73 (0.024) | -0.76 (-0.014) | -0.5 | 0.14 | 0.74 (0.044) | -0.13 |
|  |  |  |  | CONTRA | -0.26 | 0.8 (0.14) | 0.78 (2.1) | 0.61 | 0.3 | 0.14 | 0.71 (0.025) | -0.77 (-0.015) | -0.56 | 0.093 | 0.72 (0.045) | -0.17 |
|  |  |  |  | GAIT SPEED | 0.058 | -0.6 | -0.7 | -0.45 | -0.48 | -0.14 | -0.69 | 0.62 | 0.45 | -0.12 | -0.27 | 0.27 |
|  |  |  |  | FGA | 0.18 | -0.36 | -0.29 | -0.33 | -0.21 | -0.13 | -0.46 | 0.16 | 0.39 | 0.31 | 0.056 | 0.5 |
|  | Physiological (VHIT) | IPSILESIONAL | MEAN | Horizontal | 0.46 | -0.34 | -0.13 | 0.026 | -0.27 | -0.44 | -0.2 | 0.22 | 0.23 | -0.51 | -0.031 | 0.16 |
|  |  |  |  | Posterior | -0.23 | -0.34 | -0.53 | 0.3 | 0.65 | 0.69 | -0.26 | -0.043 | -0.27 | -0.18 | 0.067 | 0.38 |
|  |  |  |  | Anterior | 0.85 (160) | -0.23 | 0.25 | -0.47 | -0.29 | -0.73 (-0.42) | -0.06 | -0.0004 | 0.69 | 0.19 | -0.27 | 0.25 |
|  |  |  | STANDARD  DEVIATION | Horizontal | -0.28 | 0.55 | 0.55 | 0.68 | 0.43 | 0.42 | 0.82 (0.89) | -0.79 (-0.47) | -0.63 | 0.14 | 0.75 (1.5) | 0.028 |
|  |  |  |  | Posterior | 0.5 | -0.15 | 0.28 | 0.02 | -0.44 | -0.52 | 0.33 | 0.023 | 0.11 | 0.11 | 0.15 | 0.044 |
|  |  |  |  | Anterior | -0.26 | 0.62 | 0.58 | 0.67 | 0.55 | 0.4 | 0.77 (1.3) | -0.79 (-0.75) | -0.61 | -0.033 | 0.6 | -0.083 |
|  |  | CONTRALESIONAL | MEAN | Horizontal | 0.49 | -0.44 | -0.0035 | 0.14 | -0.22 | -0.04 | 0.66 | -0.22 | 0.044 | 0.17 | 0.33 | 0.44 |
|  |  |  |  | Posterior | 0.45 | -0.58 | -0.38 | -0.12 | -0.18 | -0.25 | -0.21 | 0.42 | 0.26 | -0.45 | -0.39 | 0.078 |
|  |  |  |  | Anterior | 0.083 | -0.16 | -0.093 | 0.48 | 0.75 (0.24) | 0.41 | 0.13 | -0.18 | -0.38 | -0.38 | -0.066 | 0.056 |
|  |  |  | STANDARD  DEVIATION | Horizontal | -0.29 | -0.44 | -0.61 | 0.4 | 0.36 | 0.62 | -0.0095 | 0.28 | -0.46 | -0.51 | -0.15 | -0.063 |
|  |  |  |  | Posterior | -0.0047 | 0.53 | 0.72 (63) | 0.66 | 0.32 | 0.11 | 0.83 (0.93) | -0.76 (-0.46) | -0.54 | 0.18 | 0.74 (1.5) | 0.013 |
|  |  |  |  | Anterior | -0.34 | -0.28 | -0.39 | 0.3 | 0.19 | 0.46 | 0.082 | 0.38 | -0.5 | -0.22 | -0.25 | -0.35 |
|  | Subjective |  |  | DHI | -0.14 | -0.0024 | -0.015 | 0.28 | -0.16 | 0.12 | 0.46 | 0.057 | -0.33 | -0.28 | 0.064 | -0.3 |
|  |  |  |  | ABC | -0.19 | 0.1 | 0.11 | -0.15 | 0.32 | 0.17 | -0.12 | -0.31 | 0.14 | 0.55 | 0.13 | 0.35 |
|  |  |  |  | HEADACHE | 0.0044 | 0.35 | 0.47 | 0.89 (0.003) | 0.53 | 0.27 | 0.74 (0.01) | -0.7 | -0.66 | -0.35 | 0.68 | 0.057 |
|  |  |  |  | BECK ANXIETY | -0.59 | 0.092 | -0.24 | 0.49 | 0.019 | 0.46 | 0.093 | 0.017 | -0.55 | -0.37 | 0.42 | -0.094 |
|  |  |  |  |  | Peak  Vel. | Cycle  Dur. | Mov.  range | Peak  Vel. | Cycle  Dur. | Mov.  range | Peak  Vel. | Cycle  Dur. | Mov.  range | Peak  Vel. | Cycle  Dur. | Mov.  range |
|  |  |  |  |  | Mean | | | CV | | | Mean | | | CV | | |
|  |  |  |  |  |  | | | | | | Asymmetry | | | | | |
|  |  |  |  |  | Kinematic Measures | | | | | | | | | | | |

| Supplementary Table 17  Correlation coefficients (slope) for Task 5 (Preop. Clinical vs. Postop. Kinematics) | | | | | | | | | | | | | | | | |
| --- | --- | --- | --- | --- | --- | --- | --- | --- | --- | --- | --- | --- | --- | --- | --- | --- |
| Clinical Measures | Functional |  | DVA | IPSI | -0.47 | 0.78 (2.8) | 0.33 | -0.39 | 0.14 | -0.48 | -0.23 | -0.21 | -0.087 | 0.34 | -0.67 | 0.4 |
|  |  |  |  | CONTRA | -0.57 | 0.67 | 0.12 | -0.24 | 0.1 | -0.34 | -0.13 | -0.45 | 0.041 | 0.033 | -0.75 (-1.7) | 0.49 |
|  |  |  | TUG | IPSI | -0.42 | 0.92 (0.22) | 0.54 | -0.14 | 0.18 | -0.51 | 0.21 | -0.46 | -0.091 | 0.46 | -0.49 | 0.37 |
|  |  |  |  | CONTRA | -0.5 | 0.95 (0.24) | 0.55 | -0.16 | 0.28 | -0.49 | 0.2 | -0.46 | -0.037 | 0.46 | -0.52 | 0.32 |
|  |  |  |  | GAIT SPEED | 0.7 | -0.77 (-1.8) | -0.37 | 0.4 | -0.45 | 0.47 | 0.019 | 0.25 | 0.017 | -0.12 | 0.79 (0.69) | -0.21 |
|  |  |  |  | FGA | 0.46 | -0.4 | -0.058 | 0.24 | -0.15 | 0.1 | 0.54 | -0.2 | 0.089 | -0.21 | 0.82 (0.056) | 0.19 |
|  | Physiological (VHIT) | IPSILESIONAL | MEAN | Horizontal | 0.75 (150) | -0.37 | 0.21 | 0.53 | -0.2 | 0.034 | -0.23 | 0.31 | -0.087 | -0.1 | 0.52 | -0.087 |
|  |  |  |  | Posterior | -0.22 | -0.22 | -0.34 | 0.21 | 0.56 | 0.63 | -0.053 | -0.38 | 0.89 (0.36) | -0.25 | 0.37 | 0.11 |
|  |  |  |  | Anterior | 0.46 | -0.23 | 0.29 | -0.34 | -0.091 | -0.57 | 0.0055 | 0.52 | -0.47 | -0.2 | 0.31 | 0.13 |
|  |  |  | STANDARD  DEVIATION | Horizontal | -0.57 | 0.82 (6.5) | 0.26 | -0.064 | 0.28 | -0.21 | -0.014 | -0.64 | 0.28 | 0.26 | -0.54 | 0.5 |
|  |  |  |  | Posterior | 0.48 | -0.053 | 0.24 | 0.62 | -0.51 | -0.29 | 0.15 | -0.2 | -0.37 | -0.39 | -0.024 | 0.26 |
|  |  |  |  | Anterior | -0.66 | 0.85 (11) | 0.3 | -0.31 | 0.46 | -0.26 | -0.21 | -0.36 | 0.25 | 0.38 | -0.63 | 0.34 |
|  |  | CONTRALESIONAL | MEAN | Horizontal | 0.31 | -0.12 | -0.21 | 0.47 | -0.48 | 0.046 | -0.44 | -0.33 | 0.11 | -0.44 | -0.1 | 0.62 |
|  |  |  |  | Posterior | 0.53 | -0.59 | -0.18 | 0.39 | -0.19 | 0.26 | -0.51 | 0.41 | 0.024 | -0.37 | 0.24 | -0.18 |
|  |  |  |  | Anterior | -0.35 | 0.027 | -0.051 | 0.11 | 0.68 | 0.26 | -0.35 | -0.15 | 0.63 | -0.35 | -0.18 | 0.045 |
|  |  |  | STANDARD  DEVIATION | Horizontal | -0.14 | -0.34 | -0.55 | 0.58 | 0.11 | 0.82 (6) | -0.59 | -0.2 | 0.68 | -0.36 | -0.2 | -0.15 |
|  |  |  |  | Posterior | -0.36 | 0.79 (6.4) | 0.48 | 0.17 | 0.2 | -0.42 | 0.22 | -0.69 | 0.084 | 0.054 | -0.47 | 0.54 |
|  |  |  |  | Anterior | -0.27 | -0.22 | -0.48 | 0.69 | -0.092 | 0.62 | -0.21 | -0.4 | 0.36 | -0.55 | -0.53 | -0.2 |
|  | Subjective |  |  | DHI | -0.048 | 0.078 | -0.2 | 0.33 | -0.36 | 0.2 | -0.59 | -0.017 | -0.039 | -0.0026 | -0.6 | -0.045 |
|  |  |  |  | ABC | -0.3 | 0.15 | 0.069 | -0.45 | 0.4 | -0.19 | 0.57 | -0.31 | 0.19 | -0.034 | 0.28 | 0.3 |
|  |  |  |  | HEADACHE | -0.26 | 0.64 | 0.4 | 0.3 | 0.41 | -0.12 | -0.27 | -0.47 | 0.4 | 0.1 | -0.35 | 0.39 |
|  |  |  |  | BECK ANXIETY | 0.0046 | 0.11 | -0.15 | 0.63 | -0.16 | 0.51 | -0.13 | -0.41 | 0.36 | 0.24 | -0.035 | -0.045 |
|  |  |  |  |  | Peak  Vel. | Cycle  Dur. | Mov.  range | Peak  Vel. | Cycle  Dur. | Mov.  range | Peak  Vel. | Cycle  Dur. | Mov.  range | Peak  Vel. | Cycle  Dur. | Mov.  range |
|  |  |  |  |  | Mean | | | CV | | | Mean | | | CV | | |
|  |  |  |  |  |  | | | | | | Asymmetry | | | | | |
|  |  |  |  |  | Kinematic Measures | | | | | | | | | | | |

| Supplementary Table 18  Correlation coefficients (slope) for Task 6 (Preop. Clinical vs. Postop. Kinematics) | | | | | | | | | | | | | | | | |
| --- | --- | --- | --- | --- | --- | --- | --- | --- | --- | --- | --- | --- | --- | --- | --- | --- |
| Clinical Measures | Functional |  | DVA | IPSI | -0.13 | 0.68 | 0.64 | -0.22 | -0.13 | -0.55 | 0.33 | -0.44 | -0.27 | -0.15 | -0.24 | -0.045 |
|  |  |  |  | CONTRA | -0.19 | 0.54 | 0.5 | -0.14 | -0.24 | -0.42 | 0.46 | -0.54 | -0.31 | -0.0029 | -0.15 | 0.19 |
|  |  |  | TUG | IPSI | -0.34 | 0.87 (0.19) | 0.79 (2.1) | 0.12 | -0.085 | -0.56 | 0.3 | -0.46 | -0.44 | -0.37 | -0.32 | -0.073 |
|  |  |  |  | CONTRA | -0.39 | 0.9 (0.2) | 0.8 (2.3) | 0.17 | 0.0066 | -0.52 | 0.27 | -0.44 | -0.46 | -0.43 | -0.35 | -0.11 |
|  |  |  |  | GAIT SPEED | 0.22 | -0.69 | -0.67 | 0.076 | -0.21 | 0.48 | -0.36 | 0.45 | 0.27 | 0.29 | 0.44 | 0.048 |
|  |  |  |  | FGA | 0.16 | -0.39 | -0.3 | 0.11 | -0.11 | 0.25 | 0.11 | -0.095 | 0.4 | 0.24 | 0.36 | 0.33 |
|  | Physiological (VHIT) | IPSILESIONAL | MEAN | Horizontal | 0.61 | -0.36 | -0.11 | 0.11 | 0.21 | 0.012 | 0.092 | 0.0069 | 0.5 | -0.091 | -0.35 | -0.58 |
|  |  |  |  | Posterior | -0.27 | -0.32 | -0.46 | 0.44 | 0.39 | 0.8 (0.38) | -0.15 | 0.089 | 0.13 | -0.16 | 0.4 | 0.018 |
|  |  |  |  | Anterior | 0.84 (120) | -0.23 | 0.15 | -0.62 | 0.16 | -0.48 | 0.45 | -0.35 | 0.83 (0.24) | 0.31 | -0.29 | -0.031 |
|  |  |  | STANDARD  DEVIATION | Horizontal | -0.42 | 0.69 | 0.55 | 0.19 | -0.11 | -0.25 | 0.31 | -0.47 | -0.45 | -0.29 | -0.1 | 0.017 |
|  |  |  |  | Posterior | 0.49 | -0.083 | 0.22 | 0.055 | -0.31 | -0.42 | 0.62 | -0.5 | 0.18 | 0.16 | -0.48 | -0.079 |
|  |  |  |  | Anterior | -0.37 | 0.73 (8.2) | 0.58 | 0.063 | 0.12 | -0.28 | 0.17 | -0.34 | -0.38 | -0.39 | -0.19 | -0.15 |
|  |  | CONTRALESIONAL | MEAN | Horizontal | 0.47 | -0.29 | -0.11 | -0.13 | -0.53 | -0.096 | 0.61 | -0.53 | 0.23 | 0.41 | 0.021 | 0.086 |
|  |  |  |  | Posterior | 0.89 | -0.6 | -0.39 | -0.053 | 0.16 | 0.21 | 0.022 | 0.14 | 0.47 | 0.12 | -0.19 | -0.41 |
|  |  |  |  | Anterior | 0.033 | -0.096 | -0.065 | 0.26 | 0.63 | 0.35 | 0.16 | -0.16 | 0.21 | -0.3 | -0.25 | -0.34 |
|  |  |  | STANDARD  DEVIATION | Horizontal | -0.2 | -0.42 | -0.56 | 0.47 | 0.098 | 0.72 (5.5) | -0.25 | 0.31 | -0.23 | -0.12 | 0.13 | -0.26 |
|  |  |  |  | Posterior | -0.17 | 0.67 | 0.71 (65) | 0.25 | -0.05 | -0.47 | 0.61 | -0.72 (-0.64) | -0.27 | -0.3 | -0.43 | -0.052 |
|  |  |  |  | Anterior | -0.32 | -0.25 | -0.36 | 0.52 | -0.11 | 0.45 | -0.051 | 0.15 | -0.5 | -0.043 | -0.084 | -0.011 |
|  | Subjective |  |  | DHI | -0.058 | 0.042 | -0.024 | 0.065 | -0.35 | -0.051 | -0.051 | 0.11 | -0.44 | -0.0011 | -0.15 | -0.23 |
|  |  |  |  | ABC | -0.18 | 0.12 | 0.09 | -0.095 | 0.14 | 0.037 | 0.18 | -0.29 | 0.21 | 0.09 | 0.32 | 0.51 |
|  |  |  |  | HEADACHE | -0.056 | 0.48 | 0.5 | 0.38 | 0.28 | -0.16 | 0.39 | -0.49 | -0.12 | -0.51 | -0.47 | -0.51 |
|  |  |  |  | BECK ANXIETY | -0.49 | 0.096 | -0.15 | 0.63 | -0.24 | 0.38 | -0.34 | 0.28 | -0.61 | -0.3 | 0.16 | -0.23 |
|  |  |  |  |  | Peak  Vel. | Cycle  Dur. | Mov.  range | Peak  Vel. | Cycle  Dur. | Mov.  range | Peak  Vel. | Cycle  Dur. | Mov.  range | Peak  Vel. | Cycle  Dur. | Mov.  range |
|  |  |  |  |  | Mean | | | CV | | | Mean | | | CV | | |
|  |  |  |  |  |  | | | | | | Asymmetry | | | | | |
|  |  |  |  |  | Kinematic Measures | | | | | | | | | | | |

| Supplementary Table 19  Correlation coefficients for Task 1 (Preop. Clinical vs. Preop. Kinematics) | | | | | | | | | | | | | | | | |
| --- | --- | --- | --- | --- | --- | --- | --- | --- | --- | --- | --- | --- | --- | --- | --- | --- |
| Clinical Measures | Functional |  | DVA | IPSI | -201.471 | 2.015 | 25.826 | 0.126 | -0.028 | -0.295 | 0.291 | -0.102 | -0.028 | -0.131 | -0.292 | 0.427 |
|  |  |  |  | CONTRA | -372.55 | 3.908 | 41.444 | 0.204 | 0.01 | -0.547 | 0.578 | -0.208 | -0.092 | 0.083 | -0.576 | 1.381 |
|  |  |  | TUG | IPSI | -12.131 | 0.112 | 1.248 | 0.014 | 0.003 | -0.014 | 0.022 | -0.01 | 0.006 | -0.018 | -0.045 | 0.035 |
|  |  |  |  | CONTRA | -14.499 | 0.114 | 1.073 | 0.015 | 0.006 | -0.011 | 0.023 | -0.01 | 0.006 | -0.018 | -0.044 | 0.032 |
|  |  |  |  | GAIT SPEED | 192.22 | -1.164 | -2.053 | -0.068 | -0.027 | 0.054 | -0.147 | 0.021 | 0.016 | -0.134 | 0.005 | -0.376 |
|  |  |  |  | FGA | 12.621 | -0.077 | -0.506 | -0.003 | -0.002 | 0.005 | -0.011 | 0.003 | 0.006 | -0.003 | -0.03 | -0.001 |
|  | Physiological (VHIT) | IPSILESIONAL | MEAN | Horizontal | 259.476 | -1.781 | -13.432 | -0.002 | -0.133 | 0.158 | -0.201 | 0.065 | 0.015 | -0.082 | 0.209 | -1.287 |
|  |  |  |  | Posterior | 80.165 | -1.278 | -12.904 | -0.02 | 0.298 | 0.28 | -0.039 | 0.029 | 0.097 | -0.049 | -0.214 | -0.85 |
|  |  |  |  | Anterior | 128.95 | -0.774 | -11.97 | -0.133 | -0.323 | 0.032 | -0.301 | 0.176 | -0.065 | 0.289 | 0.306 | -0.216 |
|  |  |  | STANDARD  DEVIATION | Horizontal | -368.194 | 3.556 | 48.15 | 0.442 | 0.369 | -0.431 | 0.834 | -0.365 | 0.152 | -0.564 | -1.544 | 0.589 |
|  |  |  |  | Posterior | 608.495 | -0.872 | -11.683 | 0.269 | -0.956 | -0.401 | -0.063 | -0.099 | -0.162 | 0.478 | -0.121 | -0.305 |
|  |  |  |  | Anterior | -775.498 | 5.321 | 61.049 | 0.554 | 0.605 | -0.413 | 1.11 | -0.375 | 0.159 | -0.725 | -1.338 | 0.01 |
|  |  | CONTRALESIONAL | MEAN | Horizontal | 265.102 | 0.165 | 24.627 | 0.054 | -0.221 | -0.344 | 0.121 | -0.094 | -0.117 | -0.03 | -0.258 | -0.574 |
|  |  |  |  | Posterior | 312.729 | -2.227 | -20.161 | -0.106 | -0.179 | 0.216 | -0.307 | 0.133 | -0.109 | 0.189 | 0.855 | -1.734 |
|  |  |  |  | Anterior | 14.109 | -1.075 | -23.355 | -0.002 | 0.183 | 0.328 | -0.059 | 0.084 | 0.01 | 0.24 | 0.327 | -1.196 |
|  |  |  | STANDARD  DEVIATION | Horizontal | 808.017 | -9.661 | -62.927 | 0.511 | 3.629 | 2.144 | 1.444 | -0.875 | -0.478 | -0.167 | 4.896 | -14.914 |
|  |  |  |  | Posterior | -189.16 | 2.468 | 17.733 | 0.499 | 0.058 | -0.299 | 0.641 | -0.283 | 0.157 | -0.167 | -1.465 | 0.428 |
|  |  |  |  | Anterior | -489.832 | 2.884 | -45.042 | 0.603 | 1.364 | 0.29 | 1.493 | -0.825 | -0.562 | 1.732 | 2.756 | 0.196 |
|  | Subjective |  |  | DHI | -0.444 | 0.011 | 0.203 | 0.001 | 0 | -0.002 | 0.002 | -0.001 | -0.001 | -0.001 | 0.003 | -0.002 |
|  |  |  |  | ABC | -0.849 | 0.001 | -0.066 | -0.001 | 0.001 | 0.001 | -0.001 | 0.001 | 0.001 | 0.001 | -0.007 | 0.009 |
|  |  |  |  | HEADACHE | 0.843 | -0.006 | -0.015 | 0.005 | 0.003 | 0.002 | 0.005 | -0.002 | 0.002 | -0.005 | -0.008 | -0.033 |
|  |  |  |  | BECK ANXIETY | 1.765 | 0 | 0.573 | 0.007 | 0.01 | -0.002 | 0.01 | -0.008 | 0.003 | -0.02 | -0.016 | -0.02 |
|  |  |  |  |  | Peak  Vel. | Cycle  Dur. | Mov.  range | Peak  Vel. | Cycle  Dur. | Mov.  range | Peak  Vel. | Cycle  Dur. | Mov.  range | Peak  Vel. | Cycle  Dur. | Mov.  range |
|  |  |  |  |  | Mean | | | CV | | | Mean | | | CV | | |
|  |  |  |  |  | Both sides | | | | | | Asymmetry | | | | | |
|  |  |  |  |  | Kinematic Measures | | | | | | | | | | | |
